# Effect of physical activity on sex hormones in women: a systematic review and meta-analysis of randomized controlled trials

**DOI:** 10.1186/s13058-015-0647-3

**Published:** 2015-11-05

**Authors:** Kaoutar Ennour-Idrissi, Elizabeth Maunsell, Caroline Diorio

**Affiliations:** Axe Oncologie, Centre de recherche du CHU de Québec-Université Laval, St-Sacrement Hospital, 1050 chemin Sainte-Foy, Quebec City, QC Canada G1S 4L8; Centre de recherche sur le cancer, Université Laval, St-Sacrement Hospital, 1050 chemin Sainte-Foy, Quebec City, QC Canada G1S 4L8; Département de médecine sociale et préventive, Faculté de médecine, Pavillon Ferdinand-Vandry, Loc 2428, Université Laval, 1050 avenue de la Médecine, Quebec City, QC Canada G1V 0A6; Centre des Maladies du Sein Deschênes-Fabia, St-Sacrement Hospital, 1050 chemin Sainte-Foy, Québec City, QC Canada G1S 4L8

## Abstract

**Introduction:**

Exposure to high levels of endogenous estrogens is a main risk factor for breast cancer in women, and in observational studies was found to be inversely associated with physical activity. The objective of the present study is to determine the effect of physical activity interventions on sex hormone levels in healthy women.

**Methods:**

Electronic databases (MEDLINE, EMBASE, CENTRAL), from inception to December 2014, and reference lists of relevant reviews and clinical trials were searched, with no language restrictions applied. Randomized controlled trials (RCTs) were included if they compared any type of exercise intervention to no intervention or other interventions, and assessed the effects on estrogens, androgens or the sex hormone binding globulin (SHBG) in cancer-free women. Following the method described in the Cochrane Handbook for Systematic Reviews of Interventions, data on populations, interventions, and outcomes were extracted, and combined using the inverse-variance method and a random-effects model. A pre-established protocol was drawn up, in which the primary outcome was the difference in circulating estradiol concentrations between the physical activity (experimental) and the control groups after intervention. Pre-specified subgroup analyses and sensitivity analysis according to the risk of bias were conducted.

**Results:**

Data suitable for quantitative synthesis were available from 18 RCTs (1994 participants) for total estradiol and from 5 RCTs (1245 participants) for free estradiol. The overall effect of physical activity was a statistically significant decrease of both total estradiol (standardized mean difference [SMD] -0.12; 95 % confidence interval [CI] -0.20 to −0.03; P = 0.01; *I*^2^ = 0 %) and free estradiol (SMD −0.20; 95 % CI −0.31 to −0.09; P = 0.0005; *I*^2^ = 0 %). Subgroup analyses suggest that this effect is independent of menopausal status and is more noticeable for non-obese women and for high intensity exercise. Meta-analysis for secondary outcomes found that physical activity induces a statistically significant decline of free testosterone, androstenedione, dehydroepiandrosterone–sulfate and adiposity markers, while a significant increase of SHBG was observed.

**Conclusions:**

Although the effect is relatively modest, physical activity induces a decrease in circulating sex hormones and this effect is not entirely explained by weight loss. The findings emphasize the benefits of physical activity for women.

**Electronic supplementary material:**

The online version of this article (doi:10.1186/s13058-015-0647-3) contains supplementary material, which is available to authorized users.

## Introduction

The practice of regular physical activity is recommended worldwide by different public health agencies [1] for primary or secondary prevention of many health problems, including cancers [2, 3]. In particular, physical activity is associated with a 25 % reduction in the average risk of breast cancer among women [4], and this protective effect appears to be independent of menopausal status [5]. Many interrelated biological mechanisms may underlie this association, such as the effect of physical activity on glucose metabolism, inflammation, immune function, and sex hormones [6]. Indeed, endogenous sex hormones, particularly estrogens, seem to be involved in the initiation, promotion, and progression of tumors [7]. Prolonged exposure to high endogenous hormone levels is considered one of the main risk factors for female breast cancer [8–10], with a relative risk of 2.00 (95 % confidence interval (CI): 1.47–2.71) for postmenopausal women with the highest estradiol levels [10]. Several observational studies have found an inverse association between physical activity and circulating estrogen levels [11, 12]. This effect may be mediated by the decrease in fat mass [13], the main source of estrogens in postmenopausal women. It may also be mediated by the disruption of the menstrual cycle before menopause [14], especially when exercise is associated with low energy intakes. However, the assessment of the exposure to physical activity remains imprecise, because it is generally only possible to measure it in naturally living subjects using questionnaires [4, 15].

Sex hormones levels could be an objective marker for evaluating the level and the effectiveness of exposure to physical activity, and for specifying the type and dose of appropriate physical activity. If physical activity is shown to have an effect on sex hormones levels, the present systematic review would also clarify one of the biological mechanisms linking physical activity to breast cancer risk. The objective of this systematic review of randomized controlled trials is to determine the effect of physical activity on healthy women’s sex hormone levels, according to the type, modality, intensity, and duration of physical activity, and according to the women’s weight and hormonal status.

## Methods

A systematic review of randomized controlled trials, with meta-analysis where possible, was performed following a pre-established protocol and in accordance with the method described in the *Cochrane Handbook for Systematic Reviews of Interventions* [16].

### Criteria for considering studies for this review

#### Types of studies

Any randomized controlled trial that assessed the effects of physical activity or exercise interventions on sex hormones in women was considered, regardless of the modality, type, intensity, or duration of the intervention. Crossover trials were considered eligible, but only the results from the first phase were eligible for inclusion in the quantitative analysis, as an intervention could lead to long-term effects. No restrictions were applied regarding the language or the publication type (articles, short reports, and abstracts).

#### Types of participants

Adult cancer-free women were eligible, regardless of hormonal group (premenopausal, perimenopausal, or postmenopausal) and body mass index (BMI; normal weight, overweight, or obese). Age, weight, and hormonal status were considered as defined by the studies. No participants were excluded based on ethnicity.

#### Types of interventions

Trials that compared a physical activity intervention with no treatment, or with any other treatments or procedures, were included. Physical activity interventions included: physical exercise of any type, modality, intensity, and duration, as well as lifestyle interventions that included exercise (i.e., exercise advice); with or without diet or other cointervention. Trials comparing more than one form of physical activity were also eligible, and if no definition was provided by the authors, the less intense intervention was considered the control group. No restriction was applied regarding who administered the intervention (physical activity professionals, health professionals, or researchers).

#### Types of outcome measures

The primary outcomes were circulating concentrations of total and free estradiol.

The secondary outcomes were circulating concentrations of the other estrogens (estrone and estriol), androgens (testosterone and its derivatives, androstenedione and dehydroepiandrosterone (DHEA)), sex hormone binding globulin (SHBG), urinary concentrations of sex steroid metabolites, anthropometric factors (weight, BMI, and other measures of adiposity), hormonal function (menstrual regularity and cycle length, or menopausal symptoms – that is, hot flushes or night sweats), and any side effects of exercise interventions.

The effect of intervention on the primary and secondary outcomes was estimated using the measurements obtained after intervention and/or the variation observed after intervention (the differences between the values obtained before and after intervention).

### Search methods for identification of studies

An electronic search of randomized controlled trials was conducted in MEDLINE (via PubMed), EMBASE, and CENTRAL (Cochrane Central Register of Controlled Trials) from inception to December 2014 (see Additional file 1: Table S1). Search strategies were developed for each of these databases with text words and index terms related to the population, intervention, and sex hormones, using appropriate filters for randomized trials on MEDLINE and EMBASE [16]. No language or publication date restrictions were applied. The reference lists of relevant reviews as well as the included studies were scanned.

### Data collection and analysis

#### Selection of studies

The references identified by the search strategy were reviewed by one author (KE-I) in a two-step process: the title and abstract of each study were screened to exclude the obviously noneligible studies; and the full text of retained articles was examined and subjected to evaluation using the predefined eligibility criteria. Whenever required, a second review author (CD) was consulted. When required, further information was sought from the authors by email.

#### Data extraction

Data extraction was performed using an exhaustive standardized and piloted form designed for this review and tested on a reference publication by one review author (KE-I). Information about the study (eligibility criteria, sample size, and methodology), participant characteristics (age, ethnicity, unhealthy behaviors, hormonal group, BMI, hormonal derivatives use, type and level of habitual physical activity, diet, and habitual energy intake), the description of the intervention, co-interventions, and comparators, and different quality evaluation criteria (sequence generation, allocation concealment, blinding of participants and personnel, blinding of outcome assessment, incomplete outcome data, selective reporting bias, and other sources of bias) were collected. The study definition of each characteristic or variable retained was recorded. In the case of multiple publications related to the same study, the publication reporting the outcomes of interest to the present review or the publication with the longer term follow-up of these outcomes was considered as the reference, and information was supplemented by secondary publications as required. The data were extracted twice over the course of several days to ensure their quality.

### Assessment of risk of bias in retained studies

The assessment of the risk of bias was performed twice by a review author (KE-I) using the Cochrane risk of bias assessment tool [16], both for the risk of bias in each study and for the overall risk of bias across studies. When required, a second reviewer (CD) was consulted. Sensitivity analyses were planned to explore the impact of different levels of risk of bias on the overall intervention effect.

### Assessment of heterogeneity

Differences between studies, including intervention characteristics (differences in modality, type, intensity, and duration), presence or absence of cointervention, and participants’ characteristics before (BMI and hormonal status) and after (weight loss) intervention, were considered for subgroup analysis.

### Measures of intervention effect and data synthesis

A quantitative synthesis of data for the comparison “Any intervention that includes physical activity” versus “Any intervention that does not include physical activity” was conducted with RevMan 5.3 software (Cochrane Review Manager Version 5.3; Nordic Cochrane Centre, Cochrane Collaboration, Copenhagen, Denmark).

The intervention effect on circulating concentrations of total and free estradiol, and most other secondary outcomes, was treated as a continuous variable. The data were extracted first as reported by the studies, and then transformed as required [16]. When required, the values were extracted from graphs using the Plot-digitizer software http://plotdigitizer.sourceforge.net/, and standard deviations were derived from standard errors or CIs using the RevMan software. The mean difference (MD) between the intervention groups was calculated using the values obtained at the end of follow-up. When the values at the end of follow-up were not reported, the value of the observed change after the intervention (MDs before–after intervention) for each group was used [16]. This combination of measures at the end of follow-up and measures of change is based on the assumption of comparability of baseline measures for the two groups provided by randomization. When the outcome was assessed at different time points during the same study, only end-of-study results were used. Either arithmetic or geometric means were used, whichever was reported by the majority of studies. For instance, the majority of studies reported arithmetic means of total estradiol, and therefore arithmetic means were used to compile data and the reported geometric means were transformed into the arithmetic form. For free estradiol, the majority of studies reported geometric means; thus, geometric means were used and the arithmetic means were transformed. The transformation from one form to the other was performed using the first method described by Higgins et al. [17]. Finally, the units were converted to those reported by the majority of studies. For the main comparison, if multiple intervention and/or control groups were compared in the same study, the respective groups were combined in pairs with RevMan software or considered separately when the distinction was relevant.

The inverse-variance method was used to combine data from different studies, using a random effects model to account for both random error and real differences in populations and interventions between studies (variability within and between studies). The overall effect was expressed as the MD between the two groups. The standardized mean difference (SMD) was used when the measurement scales between the different studies could not be corrected by the conversion into a single unit of measurement, suggesting an error in the unit reported or a difference in the methods used for measurements [16]. Statistical heterogeneity was assessed by the *I*^2^ test, with *I*^2^ > 50 % or greater indicating the presence of substantial heterogeneity [18]. The predefined subgroup analysis and sensitivity analysis were performed regardless of the value of *I*^2^. The overall risk of bias across studies and the presence of potential publication bias (funnel plot method) were evaluated [16, 19]. A statistical significance level of 5 % was applied to all analyses.

## Results

### Results of the search

**Fig 1:**
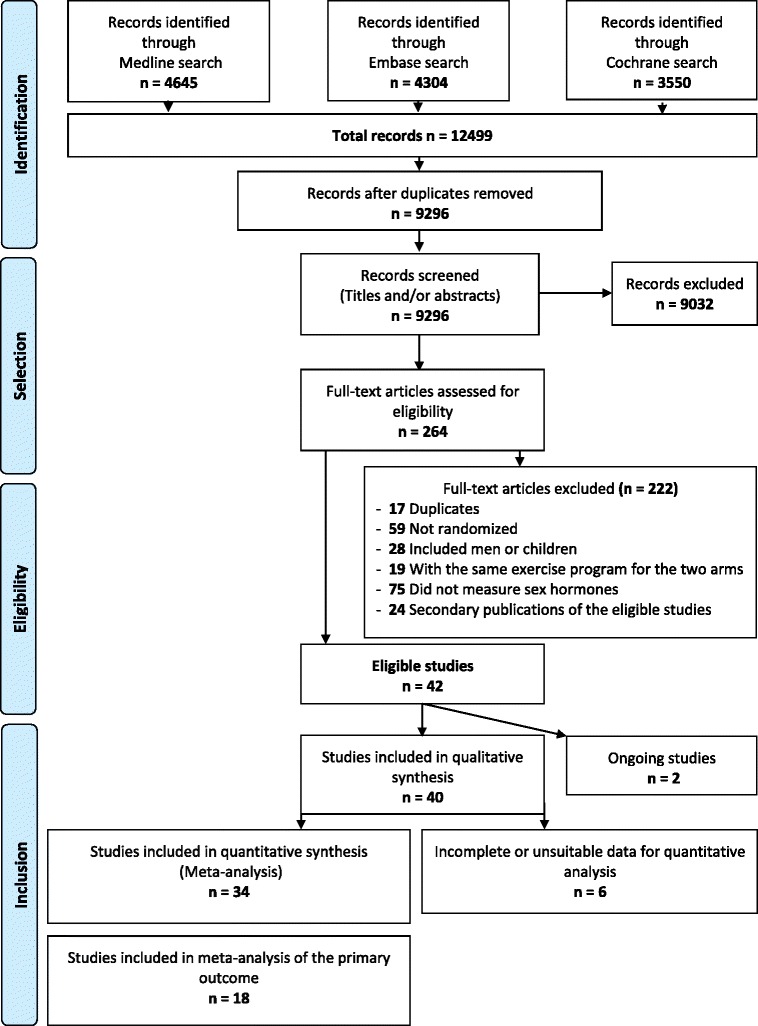
Flow diagram according to Preferred Reporting Items of Systematic Reviews and Meta-Analyses, with modifications

A total of 9296 references were retrieved by electronic search, of which 42 were eligible, including two ongoing studies [20, 21] (Fig. 1). For six studies, the data were not suitable for quantitative analysis and were not included (see Additional file 1: Table S2). Eighteen studies were included in the meta-analysis of the primary outcome, circulating estradiol concentrations.

### Description of studies

Characteristics of included studies by hormonal groups are summarized in Table 1 and are described in Table S3 (see Additional file 1). The 40 included studies were published between 1994 and 2014 and had randomized between 12 and 439 participants (median = 47 participants). Ten studies were conducted on premenopausal eumenorrheic women [22–32], one study on perimenopausal women [33], 13 studies on postmenopausal women [21, 34–53], and 12 studies on women with polycystic ovary syndrome (PCOS) [54–67]. One study did not specify the hormonal status of participants [68], and one study was conducted on perpartum women [69] and two studies on postpartum women [70, 71]. The mean age of participants varied between 15.5 and 71.0 years; the mean BMI varied between 19.9 and 39.0 kg/m^2^.

**Table 1:** Summary characteristics of eligible studies (*n* = 40)

Participants	
	– Number of randomized participants: 12–439
	– Mean age: 15.5–71.0 years
	– Mean BMI: 19.9–39.0 kg/m^2^
	– Hormonal groups:
	– Pre-menopausal: *n* = 10
	– Peri-menopausal: *n* = 1
	– Post-menopausal: *n* = 13
	– Polycystic ovary syndrome (PCOS): *n* = 12
	– Per-partum: *n* = 1
	– Post-partum: *n* = 2
	– Unspecified: *n* = 1
Interventions	
	– Type of exercise:
	– Endurance: *n* = 17
	– Resistance: *n* = 6
	– Endurance and resistance: *n* = 11
	– Yoga/Tai chi: *n* = 2
	– Free choice or education to exercise: *n* = 4
	– Modality
	– Supervision/monitoring
	– Direct or indirect supervision ± monitored exercise: *n* = 25
	– Monitored only exercise: *n* = 3
	– Mix of supervised and not supervised exercises: *n* = 4
	– No supervision: *n* = 5
	– Unknown: *n* = 3
	– Group sessions:
	– Exercise within group sessions: *n* = 5
	– Unknown: *n* = 35
	– Intensity:
	– Light: *n* = 1
	– Moderate: *n* = 9
	– High: *n* = 19
	– Unknown: *n* = 11
	– Frequency:
	– <3 days/week: *n* = 5
	– 3 to <5 days/week: *n* = 21
	– ≥5 days/week: *n* = 10
	– Unknown: *n* = 4
	– Total duration:
	– <3 months: *n* = 2
	– 3 to <6 months: *n* = 20
	– 6 to <12 months: *n* = 9
	– ≥12 months: *n* = 9
	– Co-intervention:
	– No co-intervention: *n* = 23
	– Diet: *n* = 12
	– Other: *n* = 8
Comparators	
	– No intervention: *n* = 19
	– Diet: *n* = 6
	– Other interventions: *n* = 19

*BMI* Body Mass Index, n number of studies

### Risk of bias in retained studies

Because the nature of the intervention precluded blinding participants and personnel administering the intervention, all studies were considered to be at high risk of performance bias (Additional file 1: Figure S1). Since this criterion does not discriminate between studies, it was not considered in the assessment of the overall risk of bias for each study.

Overall, the risk of bias of the included studies was unclear, with only two studies judged to be at low risk of bias [40, 41, 48, 49].

### Effects of interventions

#### Primary outcomes

**Fig. 2:**
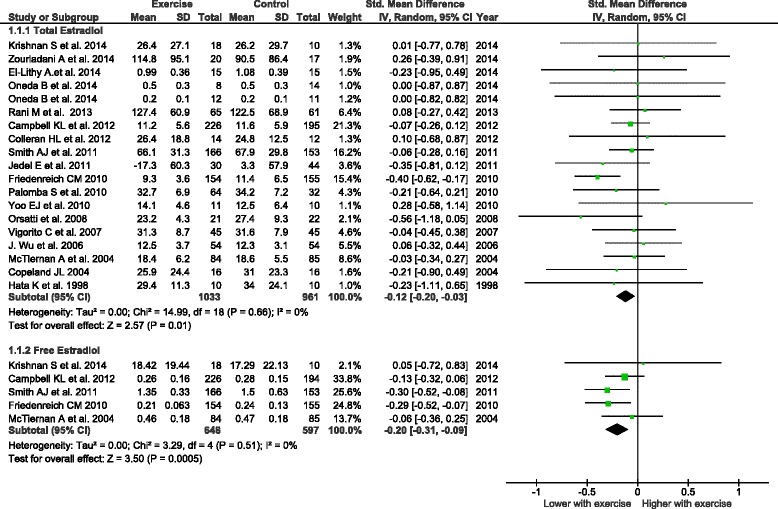
Forest plot of comparison “Any intervention that includes physical activity” vs. “Any intervention that does not include physical activity”. Outcome: total and free estradiol circulating concentrations. *CI* confidence interval, *IV* inverse variance, *SD* standard deviation, *Std. Mean Difference* standardized mean difference
- *Free estradiol*: five studies evaluated circulating concentrations of free estradiol (Fig. 2). Interventions including physical activity resulted in an overall decrease of free estradiol concentrations (*n* = 5, SMD = −0.20, 95 % CI: −0.31 to −0.09, *I*^2^ = 0 %). None of these studies included a cointervention or resistance exercise. The observed effect was more pronounced when the intervention group was compared with a nonintervention group, when the participants’ BMI was less than 30 kg/m^2^, and for interventions involving high-intensity exercise. This effect seemed to be independent of hormonal group, weight loss after intervention, amount of exercise per week, and intervention modality (see Additional file 1: Table S4). With the exclusion of Friedenreich et al.’s study [40, 41], the overall effect remained significant (SMD = −0.17, 95 % CI: −0.30 to −0.04, *I*^2^ = 0 %). However, a gap in the left bottom corner of the funnel plot graph may indicate a substantial risk of bias (Additional file 1: Figure S3).

- *Total estradiol*: 21 studies evaluated circulating concentrations of total estradiol (Fig. 2). Data from three studies [31, 42, 53] were not included in the meta-analysis (incomplete or unsuitable data; see Additional file 1: Table S2). The overall effect of interventions including physical activity was a decrease of total estradiol concentrations (*n* = 18, SMD = −0.12, 95 % CI: −0.20 to −0.03, *I*^2^ = 0 %). Subgroup analyses indicated that this effect was more pronounced when studies did not include a cointervention, when the intervention group was compared with a nonintervention group, when participants were overweight (baseline BMI between 25 and 30 kg/m^2^), and when the intervention resulted in substantial weight loss. This effect was particularly noticeable for resistance exercise, high-intensity exercise, exercise performed 3–5 hours per week, for interventions that included supervised and nonsupervised sessions, and for exercise performed in group sessions (see Additional file 1: Table S4). The only study reporting a statistically significant intervention effect was judged to be at low bias risk [40, 41], and when excluded the overall effect was no longer significant (SMD = −0.06, 95 % CI: −0.16 to 0.03, *I*^2^ = 0 %). The funnel plot was roughly symmetrical, suggesting a low risk of publication bias (Additional file 1: Figure S2).

#### Secondary outcomes

- *Other estrogens*: the overall effect on estrone circulating concentrations was not significant (*n* = 4, MD = −1.67, 95 % CI: −3.62 to 0.28, *I*^2^ = 0 %). Few studies reported the effect on the other estrogen and estrogen metabolites, and the overall effect for each of them was not significant (Table 2).

**Table 2:** Meta-analysis of secondary outcomes

Outcome or subgroup	Studies (*n*)	Participants (*n*)	Effect estimate	95 % CI	*I* ^2^ (%)
Other estrogens (mean difference)					
Estrone (pg/ml)	4	973	−1.67	−3.62, 0.28	0
Estrone sulfate (ng/ml)	2	393	−0.02	−0.19, 0.14	0
Estriol (NR)	1	79	123.90	−249.69, 497.49	NA
Estrogens – not otherwise specified (pg/ml)	2	89	27.80	−9.76, 65.36	94
Estrogen metabolites (standardized mean difference)					
2-OHE1	3	512	−0.03	−0.20, 0.15	0
16α-OHE1	3	512	0.03	−0.15, 0.20	1
Total estrogen metabolites concentration (2-OHE1 + 16α-OHE1)	1	32	0.37	−0.33, 1.07	NA
2-OHE1:16α-OHE1 ratio	2	195	−0.08	−0.36, 0.21	0
Androgens (mean difference)					
Total testosterone (ng/dl)	21	1939	−1.36	−3.83, 1.11	61
Free testosterone (pg/ml)	9	1369	−0.18	−0.29, −0.07	0
Androstenedione (pg/ml)	7	1187	–33.87	−64.44, −3.29	9
DHEA (ng/ml)	4	304	−0.08	−0.50, 0.35	0
DHEA sulfate (μmol/l)	8	697	−0.31	−0.57, −0.06	0
Sex hormone binding protein (mean difference) (nmol/l)					
	14	1634	3.93	0.98, 6.87	75
Anthropometric factors (mean difference)					
Body weight (kg)	16	1737	−1.83	−2.86, −0.81	45
Body mass index (kg/m^2^)	20	1976	−0.45	−0.87, −0.03	65
Total body fat (kg)	10	1552	−2.11	−3.71, −0.52	92
Percent fat mass (%)	13	1563	−1.28	−1.95, −0.61	54
Waist circumference (cm)	11	1274	−2.23	−2.97, −1.49	34

*CI* confidence interval, *DHEA* dehydroepiandrosterone, *NA* not applicable, *NR* not reported, *OHE1* hydroxyestrone
- *Androgens*: 26 studies reported the effect on circulating concentrations of total testosterone, of which five were not suitable for quantitative synthesis (see Additional file 1: Table S2). The funnel plot was roughly symmetrical (Figure not shown) and the overall effect was a nonstatistically significant decrease of total testosterone (*n* = 21, MD = −1.36, 95 % CI: −3.83 to 1.11, *I*^2^ = 61 %). Significant effects were observed for free testosterone (*n* = 9, MD = −0.18 pg/ml, 95 % CI: −0.29 to −0.07, *I*^2^ = 0 %), androstenedione (*n* = 7, MD = −33.87 pg/ml, 95 % CI: −64.44 to −3.29, *I*^2^ = 9 %), and DHEA sulfate (*n* = 8, MD = −0.31 μmol/l, 95 % CI: −0.57 to −0.06, *I*^2^ = 0 %). These effects seemed to be independent of hormonal group, BMI at baseline, and weight loss after intervention (see Additional file 1: Tables S5 and S6). The effect on free testosterone was slightly more pronounced for interventions with high-intensity exercise and for resistance exercise. The effect on androstenedione was more noticeable for supervised exercise (see Additional file 1: Table S6). The effect on DHEA was not significant (*n* = 4, MD = −0.08 ng/ml, 95 % CI: −0.50 to 0.35, *I*^2^ = 0 %).
- *SHBG*: 19 studies reported the effect on SHBG, of which five were not included in quantitative synthesis. The overall effect was a statistically significant increase in SHBG concentrations (*n* = 14, MD = 3.93 nmol/l, 95 % CI: 0.98–6.87, *I*^2^ = 75 %). The analysis of the different subgroups indicated that the observed effect mainly reflected the effect in the PCOS hormonal group (*n* = 8, MD = 6.76 nmol/l, 95 % CI: 5.56–7.96, *I*^2^ = 77 %) (see Additional file 1: Table S6). A gap in the bottom-left corner of the funnel plot graph was observed, indicating some risk of bias (Figure not shown).
- *Anthropometric factors*: overall, physical activity interventions resulted in statistically significant decreases in body weight, BMI, total fat mass, percent fat mass, and waist circumference (Table 2).
- *Hormonal function*: the data from the 17 studies reporting hormonal function were not suitable for quantitative synthesis. Overall, there was no significant change in menstrual cycle length of premenopausal women (3 studies) [25, 27, 28]. An improvement in the regularity or the frequency of the menstrual cycle and ovulatory parameters was observed among PCOS participants (11 studies). For postmenopausal women (two studies), there was no significant change in menopausal symptom occurrence [36, 49].
- *Side effects*: of 13 studies evaluating intervention side effects, seven did not report side effects related to exercise. One out of three studies [36, 48, 71] reported a significant decrease of bone mineral density and a nonsignificant increase of musculoskeletal injuries [36].

## Discussion

The present systematic review and meta-analysis based on randomized controlled trials conducted among healthy women demonstrates a significant decrease in total and free circulating estradiol concentrations induced by physical activity, a finding that confirms the associations reported in observational studies [11–14, 72–75]. Even though the studies reporting the effect of exercise on free estradiol represent a small part of those reporting total estradiol, the effect of exercise was more obvious for free estradiol than for total estradiol. This effect seems to be independent of menopausal status and was observed in nonobese women.

The observation that the decrease in total estradiol was related to weight loss after intervention, whereas the decrease in free estradiol was not, suggests that the effect of exercise on estradiol is not mediated solely by weight loss and may reflect the sequestration of estradiol by increasing levels of binding proteins, as was observed with SHBG levels. Similarly, there was a significant decrease in free testosterone but not in total testosterone. Except for the type of exercise, the conditions associated with the effect on total estradiol—that is, resistance exercise, high-intensity exercise, exercise performed for 3–5 hours per week—are also associated with weight loss. On the other hand, the effect of exercise modality (supervision and group sessions) indicates potential confounding and performance biases. However, it should be kept in mind that subgroup analyses are observational by nature [16] and that a major limitation for these types of interventions is the risk of performance bias. Some studies attempted to prevent this problem by administering a minimal exercise or light conditioning intervention to the comparator group. That can still be insufficient to overcome the effect of supervision and direct interaction, especially when continuous prompting is needed to maintain strenuous effort. Moreover, lack of adherence may be a problem in this type of intervention, which may negate the benefits of randomization and introduce a selection bias. In particular, the “intent-to treat” analysis is seldom really achieved and the reported values are those obtained from the participants still in the study at the end. Another problem is the social interactions introduced by group-session exercise that can potentially confound the observed associations. Therefore, the overall risk of bias within and across the included studies is unclear.

The strengths of this systematic review include the extensive and highly sensitive search strategy used to retrieve as many relevant studies as possible, and the pre-established protocol that followed procedures outlined in the *Cochrane Handbook for Systematic Reviews of Interventions* [16]. Limitations include the noninclusion of all eligible studies because of unsuitable data and the lack of high-quality evidence, as indicated by the unclear overall risk of bias. However, the observed effect on the different outcomes remains consistent despite the fact that different studies were involved for each outcome, which provides some reassurance about our conclusions. Considering that the study with the largest sample size among studies which were not included yielded nonsignificant declines in sex hormones concentrations [42], the true effect of physical activity on sex hormones might be slightly underestimated. Finally, the effect of physical activity on circulating sex hormones is relatively modest, and probably not clinically significant. The amount of circulating sex hormones may not necessarily reflect their effects on target tissues, and physical activity may still have an effect on sex hormone function by modulating target-tissue sensitivity to these hormones.

## Conclusions

In the last 20 years, numerous randomized trials with physical activity interventions targeting women have been conducted. To the best of our knowledge, this is the first systematic review and meta-analysis of such trials aimed at determining the effect of physical activity on sex hormone levels. Overall, physical activity induces a decrease in circulating sex hormones. Although different studies were involved for the different outcomes we considered, the results for overall meta-analysis and subgroup analyses were consistent. Even if it appears that the effect of physical activity is not completely accurately reflected by measures of circulating sex hormones, physical activity is a safe intervention with undeniable benefits for women, regardless of menopausal status and weight loss induced by exercise.

## Additional file

Additional file 1: Table S1. Presenting search strategies; **Table S2.** Presenting studies not included in quantitative synthesis; **Table S3.** presenting characteristics of eligible studies by hormonal group; **Table S4.** presenting subgroup analyses of primary outcomes; **Table S5.** presenting subgroup analyses of secondary outcomes: total and free testosterone; **Table S6.** presenting subgroup analyses of secondary outcomes: androstenedione, DHEA sulfate, and SHBG; **Figure S1.** showing risk of bias graph: review authors’ judgments about each risk of bias item presented as percentages across all included studies; **Figure S2.** showing funnel plots for the comparison “Any exercise intervention versus no exercise intervention”, outcome: total estradiol; and **Figure S3.** showing funnel plots for the comparison “Any exercise intervention versus no exercise intervention”, outcome: free estradiol. (DOCX 549 kb)

## Abbreviations

BMI: Body mass index
CI: Confidence interval
DHEA: Dehydroepiandrosterone
MD: Mean difference
PCOS: Polycystic ovary syndrome
SHBG: Sex hormone binding globulin
SMD: Standardized mean difference
