# Supplementary material for: Effect of physical activity on sex hormones in women: a systematic review and meta-analysis of randomized controlled trials
Source: Breast Cancer Res. 2015 Nov 5;17:139. doi: 10.1186/s13058-015-0647-3 (PMC4635995; doi:10.1186/s13058-015-0647-3)
Supplement: Additional file 1: Table S1. — Presenting search strategies; Table S2. Presenting studies not included in quantitative synthesis; Table S3. presenting characteristics of eligible studies by hormonal group; Table S4. presenting subgroup analyses of primary outcomes; Table S5. presenting subgroup analyses of secondary outcomes: total and free testosterone; Table S6. presenting subgroup analyses of secondary outcomes: androstenedione, DHEA sulfate, and SHBG; Figure S1. showing risk of bias graph: review authors’ judgments about each risk of bias item presented as percentages across all included studies; Figure S2. showing funnel plots for the comparison “Any exercise intervention versus no exercise intervention”, outcome: total estradiol; and Figure S3. showing funnel plots for the comparison “Any exercise intervention versus no exercise intervention”, outcome: free estradiol. (DOCX 549 kb) [file 13058_2015_647_MOESM1_ESM.docx]

**ADDITIONAL MATERIAL**

**Table S1:** Search strategies

**Table S2:** Studies not included in quantitative synthesis

**Table S3:** Characteristics of eligible studies by hormonal group

**Table S4**: Subgroup analyses of primary outcomes

**Table S5**: Subgroup analyses of secondary outcomes : Total and free testosterone

**Table S6:** Subgroup analyses of secondary outcomes : Androstenedione, DHEA-sulfate, SHBG

**Figure S1**: Risk of bias graph: review authors' judgements about each risk of bias item presented as percentages across all included studies

**Figure S2**: Funnel plots of comparison “Any exercise intervention versus no exercise intervention”, outcome: Total Estradiol

**Figure S3:** Funnel plots of comparison “Any exercise intervention versus no exercise intervention”, outcome: Free Estradiol

| **Table S1 - Search strategies** |
| --- |
| **MEDLINE (via PubMed)** |
| 1. "Women"[Mesh] OR females[tiab] OR female[tiab] OR women[tiab] OR Woman[tiab] 2. "Physical activity" [tiab] OR "Physically active" [tiab] OR Exercise[tiab] OR Exercises[tiab] OR Exertion[tiab] OR Walking[tiab] OR Running[tiab] OR Jogging[tiab] OR Swimming[tiab] OR Cycling[tiab] OR Bicycling[tiab] OR gymnastic[tiab] OR gymnastics[tiab] OR gym[tiab] OR "weight lift" [tiab] OR dance[tiab] OR dancing[tiab] OR Strength[tiab] OR Strengthening[tiab] OR Resistance[tiab] OR endurance[tiab] OR performance[tiab] OR Circuit[tiab] OR training[tiab] OR sport[tiab] OR sports[tiab] OR sportive[tiab] OR fitness[tiab] OR Aerobic[tiab] OR Aerobics[tiab] OR yoga[tiab] OR Tai Ji[tiab] OR Tai chi[tiab] OR "Motor activity" [tiab] OR "energy expenditure" [tiab] OR "Weight Loss" [tiab] OR muscle[tiab] OR ((behavior[tiab] OR behaviors[tiab] OR behavioural[tiab] OR behaviour[tiab] OR behaviours[tiab] OR behavioural[tiab] OR life style[tiab] OR lifestyle[tiab]) AND (change[tiab] OR changes [tiab] OR modification[tiab] OR modifications[tiab] OR intervention[tiab] OR interventions[tiab] OR therapy[tiab] OR therapies[tiab])) OR (Health[tiab] AND (Education[tiab] OR Promotion[tiab] OR behaviour[tiab] OR Behavior[tiab])) OR "Physical Conditioning, Human"[Mesh] OR "Motor Activity"[Mesh:noexp] OR "Exercise Movement Techniques"[Mesh] OR "Sports"[Mesh] OR "Exercise Therapy"[Mesh] OR "Exercise"[Mesh] 3. biomarker[tiab] OR biomarkers[tiab] OR "biological marker" [tiab] OR "biological markers" [tiab] OR biology[tiab] OR "biological effect"[tiab] OR "sex hormone" [tiab] OR "sex hormones"[tiab] OR hormonal[tiab] OR "Sex Steroid" [tiab] OR "sex steroids"[tiab] OR Steroidal[tiab] OR Gonad[tiab] OR Gonads[tiab] OR Gonadal[tiab] OR Oestrogen[tiab] OR Estrogen[tiab] OR Oestrogens[tiab] OR Estrogens[tiab] OR Oestrogene[tiab] OR Estrogene[tiab] OR Oestrogenes[tiab] OR Estrogenes[tiab] OR Estradiol[tiab] OR oestradiol[tiab] OR estriol[tiab] OR oestriol[tiab] OR estrone[tiab] OR oestrone[tiab] OR Progestogen[tiab] OR Progestogene [tiab] OR Progestogens[tiab] OR Progestogenes[tiab] OR Progesterone[tiab] OR Androgen[tiab] OR Androgene[tiab] OR Androgens[tiab] OR Androgenes[tiab] OR Androstenedione[tiab] OR Dehydroepiandrosterone[tiab] OR "DHEA" [tiab] OR Dihydrotestosterone[tiab] OR Testosterone[tiab] OR "Sex Hormone Binding Globulin" [tiab] OR "SHBG" [tiab] OR "Gonadal Hormones"[Mesh] OR "Androgens"[Mesh] OR "Estrogens"[Mesh] OR "Gonadal Steroid Hormones"[Mesh] OR "Sex Hormone-Binding Globulin"[Mesh] 4. (randomized controlled trial [Publication Type] OR controlled clinical trial [Publication Type] OR randomized [tiab]OR placebo [tiab] OR randomly [tiab] OR trial [tiab] OR groups [tiab]) NOT (animals [mh] NOT humans [mh]) 5. 1 AND 2 AND 3 AND 4 |
| **EMBASE** |
| 1. woman:ab,ti OR women:ab,ti OR Female:ti,ab OR Females:ti,ab OR 'female'/de 2. 'physical activity':ab,ti OR 'physically active':ab,ti OR Exercise:ti,ab OR Exercises:ti,ab OR exertion:ab,ti OR walking:ab,ti OR Running:ti,ab OR jogging:ti:ab OR Swimming:ti:ab OR Cycling:ti:ab OR Bicycling:ti:ab OR gymnastic:ti,ab OR gymnastics:ti,ab OR gym:ti,ab OR 'weight lift':ab,ti OR dance:ti:ab OR dancing:ti:ab OR Strength:ti,ab OR Strengthening:ti,ab OR Resistance:ti:ab OR Circuit:ti:ab OR endurance:ab,ti OR performance:ti,ab OR training:ti,ab OR sport:ti:ab OR sports:ti,ab OR sportive:ti,ab OR Fitness:ti:ab OR Aerobic:ti,ab OR Aerobics:ti,ab OR yoga:ti:ab OR 'tai ji':ab,ti OR 'tai chi':ti,ab OR 'Motor activity':ti,ab OR 'Weight Loss':ti,ab OR 'energy expenditure':ti,ab OR muscle:ti,ab OR ((lifestyle:ti:ab OR 'Life style':ti,ab OR behaviour:ti,ab OR behaviours:ti,ab OR behavioural:ti,ab OR behavior:ti,ab OR behaviors:ti,ab OR behavioral:ti,ab ) AND (therapy:ti,ab OR therapies:ti,ab OR intervention:ti,ab OR interventions:ti,ab OR modification:ti,ab OR modifications:ti,ab OR change:ti,ab OR changes:ti,ab)) OR (health:ti:ab AND (Behavior:ti,ab OR behaviour:ti,ab OR Promotion:ti,ab OR Education:ti,ab)) OR 'physical activity'/exp OR 'exercise'/exp 3. biomarker:ti,ab OR biomarkers:ti,ab OR 'biological marker':ti,ab OR 'biological markers':ti,ab OR biology:ti,ab OR 'biological effect':ti,ab OR 'sex hormone':ti,ab OR 'sex hormones':ti,ab OR hormonal:ti,ab OR 'sex steroid':ti,ab OR 'sex steroids':ti,ab OR Steroidal:ti,ab OR Gonad:ti,ab OR Gonads:ti,ab OR Gonadal:ti,ab OR Oestrogen:ti,ab OR Estrogen:ti,ab OR Oestrogens:ti,ab OR Estrogens:ti,ab OR Oestrogene:ti,ab OR Estrogene:ti,ab OR Oestrogenes:ti,ab OR Estrogenes:ti,ab OR Estradiol:ti,ab OR oestradiol:ti,ab OR estriol:ti,ab OR oestriol:ti,ab OR estrone:ti,ab OR oestrone:ti,ab OR Progestogen:ti,ab OR Progestogene:ti,ab OR Progestogens:ti,ab OR Progestogenes:ti,ab OR Progesterone:ti,ab OR Androgen:ti,ab OR Androgene:ti,ab OR Androgens:ti,ab OR Androgenes:ti,ab OR Androstenedione:ti,ab OR Dehydroepiandrosterone:ti,ab OR DHEA:ti,ab OR Dihydrotestosterone:ti,ab OR Testosterone:ti,ab OR 'Sex Hormone Binding Globulin':ti,ab OR 'SHBG':ti,ab OR 'sex hormone binding globulin'/exp OR 'sex hormone'/exp 4. random$:ti,ab OR factorial$:ti,ab OR crossover$:ti,ab OR 'cross over$':ti,ab OR cross-over$:ti,ab OR placebo$:ti,ab OR 'doubl$ adj blind$':ti,ab OR 'singl$ adj blind$':ti,ab OR assign$:ti,ab OR allocat$:ti,ab OR volunteer$:ti,ab OR 'crossover procedure'/exp OR 'double blind procedure'/exp OR 'randomized controlled trial'/exp OR 'single blind procedure'/exp 5. 1 AND 2 AND 3 AND 4 |
| **CENTRAL (Cochrane Central Register of Controlled Trials)** |
| 1. Woman:ti,ab or Women:ti,ab or Female:ti,ab or Females:ti,ab or [mh Women] 2. "Physical activity":ti,ab or "Physically active":ti,ab or Exercise:ti,ab or Exercises:ti,ab or Exertion:ti,ab or Walking:ti,ab or Running:ti,ab or Jogging:ti,ab or Swimming:ti,ab or Cycling:ti,ab or Bicycling:ti,ab or gymnastic:ti,ab or gymnastics:ti,ab or gym:ti,ab or "weight lift":ti,ab or dance:ti,ab or dancing:ti,ab or strength:ti,ab or Strengthening:ti,ab or Resistance:ti,ab or Circuit:ti,ab or endurance:ti,ab or performance:ti,ab or training:ti,ab or sport:ti,ab or sports:ti,ab or sportive:ti,ab or Fitness:ti,ab or Aerobic:ti,ab or Aerobics:ti,ab or yoga:ti,ab or "Tai Ji":ti,ab or "tai chi":ti,ab or [mh "Motor Activity"] this term only or "Weight Loss":ti,ab or "energy expenditure":ti,ab or muscle:ti,ab or ((lifestyle:ti,ab or "Life style":ti,ab or behaviour:ti,ab or behaviours:ti,ab or behavioural:ti,ab or behavior:ti,ab or behaviors:ti,ab or behavioural:ti,ab) and (therapy:ti,ab or therapies:ti,ab or intervention:ti,ab or interventions:ti,ab or modification:ti,ab or modifications:ti,ab or change:ti,ab or changes:ti,ab)) or (Health:ti,ab and (Behavior:ti,ab or behaviour:ti,ab or Promotion:ti,ab or Education:ti,ab)) or [mh Exercise] or [mh "Exercise Therapy"] or [mh Sports] or [mh "Exercise Movement Techniques"] or [mh "Motor Activity"] or [mh "Physical Conditioning, Human"] 3. biomarker:ti,ab OR biomarkers:ti,ab OR "biological marker":ti,ab OR "biological markers":ti,ab OR biology:ti,ab OR "biological effect":ti,ab OR "sex hormone":ti,ab OR "sex hormones":ti,ab OR hormonal:ti,ab OR "sex Steroid":ti,ab OR "sex steroids":ti,ab OR Steroidal:ti,ab OR Gonad:ti,ab OR Gonads:ti,ab OR Gonadal:ti,ab OR Oestrogen:ti,ab OR Estrogen:ti,ab OR Oestrogens:ti,ab OR Estrogens:ti,ab OR Oestrogene:ti,ab OR Estrogene:ti,ab OR Oestrogenes:ti,ab OR Estrogenes:ti,ab OR Estradiol:ti,ab OR oestradiol:ti,ab OR estriol:ti,ab OR oestriol:ti,ab OR estrone:ti,ab OR oestrone:ti,ab OR Progestogen:ti,ab OR Progestogene:ti,ab OR Progestogens:ti,ab OR Progestogenes:ti,ab OR Progesterone:ti,ab OR Androgen:ti,ab OR Androgene:ti,ab OR Androgens:ti,ab OR Androgenes:ti,ab OR Androstenedione:ti,ab OR Dehydroepiandrosterone:ti,ab OR DHEA:ti,ab OR Dihydrotestosterone:ti,ab OR Testosterone:ti,ab OR "Sex Hormone Binding Globulin":ti,ab OR SHBG:ti,ab OR [mh "Gonadal Hormones"] OR [mh "Androgens"] OR [mh "Estrogens"] OR [mh "Gonadal Steroid Hormones"] OR [mh "Sex Hormone-Binding Globulin"] 4. #1 and #2 and #3 |

| **Table S2 - Studies not included in quantitative synthesis** | | | |  |
| --- | --- | --- | --- | --- |
| **Reference** | **Participants (n)** | | **Result** | **Reason of non-inclusion** |
| Carpenter SE et al. 1995[^31^](#_ENREF_31) | 39 | | Non-significant increase in estradiol and SHBG, and decrease in free testosterone;  Significant decrease in testosterone | Number of participants per group not reported |
| Thomson RL et al. 2008^62,63^ | 52 | | Non-significant effect on testosterone; not-significant increase in SHBG | Reports « *model predicted values »* |
| Monninkhof EM et al. 2009[^42^](#_ENREF_42)^,^[^43^](#_ENREF_43) | 189 | | Non-significant decline of estradiol, and testosterone | Dispersion measures not reported |
| Figueroa A et al. 2003[^53^](#_ENREF_53) | 84 | | Non-significant changes in oestrone, estradiol and androstenedione | Values not reported |
| Brown AJ et al. 2009[^60^](#_ENREF_60) | 20 | | Non-significant decline in free testosterone | Reports median % change |
| Hoeger KM et al. 2004[^66^](#_ENREF_66) | 38 | | Significant decrease in androgens | Reports % change |
| SHBG : Sex hormone-binding globulin | |  |  |  |

| **Table S3 - Characteristics of eligible studies by hormonal group** | | | | | | | | | | | | | | | | | |
| --- | --- | --- | --- | --- | --- | --- | --- | --- | --- | --- | --- | --- | --- | --- | --- | --- | --- |
| **Study references**  **Year**  **Country** | | **Population** | | | **Exercise Group(s) (n)** | | | | | | **Comparator Group(s)**  **(n)** | | **Outcomes** | | | | |
|  |  | **N** | **Inclusion criteria** | **Participants**  **(mean ± SD)** | **Type and modality** | **Duration Frequency** | **Intensity** | **Duration**  **of study** | **Co-intervention** | |  |  | **Study primary outcome(s)** | **Hormones of interest** | **Adiposity markers** | **Hormonal function** | **Side effects** |
| **Pre-menopausal** | | | | | | | | | | | | | | | | | |
| \|  \|  \|  \|  \|  \|  \|  \| El-Lithy A  et al.[^22^](#_ENREF_22)  2014  Egypt \|  \|  \|  \|  \|  \|  \| \| --- \| --- \| --- \| --- \| --- \| --- \| --- \| --- \| --- \| --- \| --- \| --- \| --- \| --- \| | | **30** | Premenstrual  syndrome  Regular cycles  Age: 16-20 years  Sedentary  No OC | Age: 18.0 ± 1.5 years  BMI: 22.8 ± 2.7 kg/m^2^ | Aerobic exercise  (**n = 15**) | 25 min  3 d/wk. | - | 3 months | Vitamin B6 and calcium supplements | | Vitamin B6 and calcium supplements  No exercise  (**n = 15**) | | Premenstrual syndrome, haematological and hormonal parameters | Estradiol | - | - | - |
| Rani M  et al.[^23^](#_ENREF_23)  2013  India | | **150** | Menstrual irregularities  ± hormonal treatment | Age: 28.1 ± 7.4 years  BMI: 22.6 ± 4.6 kg/m^2^ | Yoga *Nidra*  Supervised  **(n = 65/75)** | 35-40 min  5 d/wk. |  | 6 months | Conventional treatment | | Conventional treatment  Maintain usual activities  **(n = 61/75)** | | Hormonal profile | Estradiol  Testosterone  DHEA-S | - | - | - |
| Moghadasi M et al.[^24^](#_ENREF_24)  2013  Iran | | **20** | Young healthy women  Regular  menstruation  Sedentary  No medications with effect on bone metabolism | Age: 25.3 ± 3.2 years  BMI: 23.4 ± 3.4 kg/m^2^ | Resistance exercise  Supervised  **(n = 9/10)** | 50-60 min  3 d/wk. | 65-80 % of 1-RM | 12 weeks | - | | No exercise  **(n = 10/10)** | | Growth factors and hormones | Estrogens  Testosterone | Weight  BMI  % body fat | - | - |
| Smith AJ  et al.[^25^](#_ENREF_25)^,^[^26^](#_ENREF_26)  2011  (WISER study)  USA | | **391** | Eumenorrheic  Age: 18-30 years  BMI:  18 à 40 kg/m^2^  Sedentary  No OC | Age: 25.3 ± 3.4 years  BMI: 24.7 ± 4.7 kg/m^2^ | Aerobic exercise  Supervised  **(n =** **166/212)** | 30 min  5 d/wk. | 60-85 %  HR max | 14–18 weeks  (4 menstrual cycles) | **-** | | No exercise  **(n = 153/179)** | | Sex hormones and SHBG | Estradiol  Oestrone sulfate  Testosterone  SHBG  Estrogen urinary metabolites | Weight  BMI  Fat Mass  % body fat | Menstrual cycle length | - |
| Williams NI  et al.[^27^](#_ENREF_27)  2010  USA | | **47** | Eumenorrheic  Age: 25-40years  BMI:  18–35 kg/m^2^  Sedentary  No OC | Age: 32.5 ± 4.4 years  BMI: 23.7 ± 3.0 kg/m^2^ | Aerobic exercise  Supervised  **(n = 24/36)** | 40-90 min  4 d/wk. | 79 ± 0.7 % HR max | 4 menstrual cycles | Caloric restriction  (20–35% reduction) | | Light conditioning  36 ± 2.9 min  1–2 d/wk.  77 ± 0.8 %  HR max  **(n = 9/11)** | | Menstrual cyclicity and ovarian steroids | SHBG  Estradiol (AUC)  Urinary metabolites (AUC) | Weight  Fat Mass  % body fat | Menstrual cycle length  Menstrual irregularities | - |
| Campbell  KL et al.[^28^](#_ENREF_28)  2007  Canada | | **32** | Premenopausal  Eumenorrheic  Age: 20-35 years  BMI:  18 to 29.9 kg/m^2^  Sedentary  No OC | Age: 25.7 ± 5.1 years  BMI: 22.9 ± 2.8 kg/m^2^ | Aerobic  Supervised  **(n = 17/17)** | 30-45 min  4 d/wk. | Moderate to vigorous | 12 weeks | **-** | | Maintain usual activities  **(n = 15/15)** | | Estrogen urinary metabolites | 2-OHE1  16α-OHE1  Total EMC | Weight  BMI  Waist circumference  Fat mass  % body fat | Ovulatory status | Side effects (un-specified) |
| Häkkinen K et al.[^29^](#_ENREF_29)  2002  Finland | | **21** | Premenopausal  Fibromyalgia dg  Physically active | Age: 38.1 ± 5.5 years  Weight: 70.9 ± 12.9 kg | Resistance  Supervised  **(n = 11)** | 3-4series of 10 to 20 repetitions  2 d/wk. | 40-80 %  of 1-RM | 21  weeks | **-** | | Maintain usual activities  **(n = 10)** | | Anabolic  hormones | Testosterone  (total + free)  DHEA-S | Weight  % body fat | - | - |
| Hata K  et al. [^30^](#_ENREF_30)  1998  Japan | | **20** | Eumenorrheic | Age: 23.2 ± 1.5 years  BMI: 19.9 ± 1.4 kg/m^2^ | Aerobic (dance)  Supervised  **(n = 10)** | 35 min  3 to 5 d/wk. | >60 %  HR max | 12 months | **-** | | No exercise  **(n = 10)** | | Cerebro-vascular Tone | Estradiol | - | - | - |
| **Table S3 - Characteristics of eligible studies by hormonal group (continued)** | | | | | | | | | | | | | | | | | |
| **Study references**  **Year**  **Country** | | **Population** | | | **Exercise Group(s) (n)** | | | | | | **Comparator Group(s)**  **(n)** | | **Outcomes** | | | | |
|  |  | **N** | **Inclusion criteria** | **Participants**  **(mean ± SD)** | **Type and modality** | **Duration Frequency** | **Intensity** | **Duration**  **of study** | **Co-intervention** | |  |  | **Study primary outcome(s)** | **Hormones of interest** | **Adiposity markers** | **Hormonal function** | **Side effects** |
| **Pre-menopausal** | | | | | | | | | | | | | | | | | |
| Carpenter SE et al. [^31^](#_ENREF_31)  1995  USA | | **39** | Eumenorrheic  Endometriosis  No regular exercise  No hormonal treatment | Age: 29.4 *vs* 31.0 years  Weight: 139 lbs *vs* 137 lbs | Free  (based on individualised prescriptions)  **(n = NR)** | 40 min  4 d/wk. | 50-70 %  HR max | 24 weeks | Danazol | | Danazol  Maintain usual activities  **(n = NR)** | | Danazol side Effects | Estradiol  Testosterone  SHBG | - | - | Danazol  side effects |
| Libardi CA  et al.[^32^](#_ENREF_32)  2013  Brazil | | **17** | Young healthy  women  Taking OC | Age: 23.8 ± 3.5 years  BMI: 21.9 ± 2.1 kg/m^2^ | Resistance (isokinetic dynamometer)  Supervised  **(n = 8)** | 5 series of 6 eccentric arm contractions | Fast velocity  contractions 210°/s | 30  minutes | - | | Same exercise with slow velocity contractions 30°/s  **(n = 9)** | | Acute hormonal responses | Testosterone (total + free) | - | - | - |
| **Peri-menopausal** | | | | | | | | | | | | | | | | | |
| Krishnan S  et al.[^33^](#_ENREF_33)  2014  USA | | **36** | Peri-menopausal  Menstrual  irregularities  Age: 42-52 years  BMI:  18,5-32,0 kg/m²  Sedentary / light  activity  No OC or  hormonal  treatment | Age: 46.7 ± 3.3 years  BMI: 26.6 ± 3.8 kg/m^2^ | Aerobic and resistance  Supervised  **(n = 18/21)** | 60 min  6 d/wk. | 50-80 % HR max | 6 months | - | Maintain usual activities  **(n = 10/14)** | | | Circulating androgens  Indices of insulin sensitivity/ resistance | Estradiol  Testosterone  DHEA  DHEA-S | Weight  BMI  Body fat  total  gynoid  android | Menstrual cycle length | - |
| Oneda B  et al.[^34^](#_ENREF_34)  2014  Brazil | | **60** | Post-menopausal  Hysterectomy  Age: 45-60 years  BMI: <30 kg/m^2^  Sedentary  No OC or  hormonal  treatment | Age: 50.7 ± 3.3 years  BMI: 25.6 ± 2.8 kg/m^2^ | Aerobic  exercise  Monitored | 20-50 min  3 d/wk. | Moderate | 6 months | 1/ Estradiol  (**n = 8/15**)  2/ Placebo  (**n = 12/15**) | Maintain usual activities  1/ Estradiol  (**n = 14/15**)  2/ Placebo  (**n = 11/15**) | | | Sympathetic activity  Hemo-dynamics | Estradiol | Weight  BMI | - | - |
| **Post-menopausal** | | | | | | | | | | | | | | | | | |
| van Gemert WA et al.[^21^](#_ENREF_21)  2013  (SHAPE-2 study)  Netherlands | | **243** | Post-menopausal  Age: 50-69 years  BMI:  25–35 kg/m^2^  Sedentary  No hormonal treatment | -  (ongoing study) | Endurance and strength training  Group supervised exercise +  individual home-based exercise  **(n = 98)** | 45-50 min  2 d/wk.  +  2 h/wk. | High-intensity  +  60-65 % HR max | 16 weeks | Moderate caloric restriction  (250 kcal/d) | | 1/ Caloric restriction 500kcal/d)  **(n = 97)**  2/ Control  **(n = 48)** | | Estradiol  Oestrone  Testosterone  SHBG | Estradiol  Oestrone  Testosterone  SHBG | Weight  BMI  Waist  circumference  Body fat | - | - |
| Kim JW  et al.[^35^](#_ENREF_35)  2012  South Korea | | **30** | Post-menopausal  Obese:  >32 % body fat  Sedentary  No hormonal  treatment | Age: 54.5 ± 2.8 years  BMI: 25.1 ± 1.4 kg/m^2^ | Aerobic (dance)  Group Supervised  **(n = 15)** | 60 min  3 d/wk. | 55-65 % HR max  Gradual ↑ 5%/4wk.  70-80 % HR max | 16 weeks | - | | No exercise  **(n = 15)** | | SHBG  Body fat index  Metabolic syndrome factors | SHBG | Weight  BMI  Waist circumference  % body fat | - | - |
| **Table S3 - Characteristics of eligible studies by hormonal group (continued)** | | | | | | | | | | | | | | | | | |
| **Study references**  **Year**  **Country** | | **Population** | | | **Exercise Group(s) (n)** | | | | | | **Comparator Group(s)**  **(n)** | | **Outcomes** | | | | |
|  |  | **N** | **Inclusion criteria** | **Participants**  **(mean ± SD)** | **Type and modality** | **Duration Frequency** | **Intensity** | **Duration**  **of study** | **Co-intervention** | |  |  | **Study primary outcome(s)** | **Hormones of interest** | **Adiposity markers** | **Hormonal function** | **Side effects** |
| **Post-menopausal** | | | | | | | | | | | | | | | | | |
| Campbell KL et al.[^36^](#_ENREF_36)^,[37](#_ENREF_37)^  2012  (NEW study)  Canada | | **439** | Post-menopausal  Age: 50-75 years  BMI: ≥25.0 kg/m^2^  ≥23.0 kg/m^2^ if Asian-American  Sedentary  No hormonal treatment | Age: 58.0 ± 5.0 years  BMI: 30.9 ± 4.0 kg/m^2^ | Aerobic  ≥3 group supervised sessions  2 sessions at home | ≥45 min  5 d/wk. | 70-85 %  HR max | 12 months | 1/ Exercise only  **(n = 110/117)**  2/ Calorie-reduced diet  1200 to 2000 kcal/d  **(n = 116/117)** | | 1/ Control  (delayed  Intervention)  (**n = 80/87**)  2/ Calorie-reduced diet  1200 to 2000 kcal/d  **(n = 115/118)** | | Oestrone | Oestrone  Estradiol (total+ free)  Testosterone (total+free)  Androstenedione  SHBG | Weight  BMI  Waist  circumference  % body fat | Hot flash symptoms | Musculo-skeletal injuries  Total bone mineral density |
| Tartibian B et al.[^38^](#_ENREF_38)  2011  Iran | | **79** | Post-menopausal  Sedentary  No medications | Age: 60.8 ± 6.6 years  BMI: 26.9 ± 5.5 kg/m^2^ | Aerobic (walking or jogging)  Monitored | 40-45 min  4-6 d/wk. | 55-65 % HR max | 24  Weeks | 1/ Exercise only  **(n = 18/20)**  2/ N-3 PUFA Supplement  **(n = 20/21)** | | 1/ N-3 PUFA Supplement  **(n = 18/20)**  2/ Control  **(n = 14/ 18)** | | Bone mineral density  Inflammatory markers | Estrogen | - | - | - |
| Yoo EJ  et al.[^39^](#_ENREF_39)  2010  South Korea | | **28** | Age >65 years  No hormonal  treatment | Age: 71.0 ± 2.6 years  BMI: 26.0 ± 2.9 kg/m^2^ | Weight-bearing walking exercises Supervised  (**n = 11/14**) | 45 min  3 d/wk. | 60 %  HR max | 3 months | - | | Maintain usual activities  **(n = 10/14)** | | Falls  Bone metabolism | Estradiol  Testosterone | Weight  BMI  Body fat | - | - |
| Friedenreich  CM et al.[^40^](#_ENREF_40)^,[41](#_ENREF_41)^  2010  (ALPHA trial)  Canada | | **320** | Post-menopausal  Age: 50 -74 years  BMI: 22-40 kg/m^2^  Sedentary  No hormonal  treatment | Age: 60.9 ± 5.5 years  BMI: 29.6 ± 4.4 kg/m^2^ | Aerobic  ≥3 supervised  ≤2 at home  (**n = 160)** | 45 min  5 d/wk. | 50-60 %  HR max  Gradual ↑  70-80 %  HR max | 12 months | - | | Maintain usual activities  **(n = 159/160)** | | Oestrone  Estradiol  Testosterone  Androstenedione  SHBG | Oestrone  Estradiol  Testosterone Androstenedione  SHBG | Weight  Waist circumference  BMI  Body fat  % body fat | - | Side effects (un-specified) |
| Monninkhof EM et al.[^42^](#_ENREF_42)^,[43](#_ENREF_43)^  2009  (SHAPE trial)  Netherlands | | **189** | Post-menopausal  Age: 50-69 years  BMI >22 kg/m^2^  Sedentary  No hormonal treatment | Age: 58.6 ± 4.4 years  BMI: 26.9 ± 3.2 kg/m^2^ | Aerobic and resistance group exercises  supervised  + at home  **(n = 94/96)** | 45 min  3 d /wk.  + 30 min/wk. | 60-85 % HR max | 12 months | - | | Maintain usual activities  **(n = 88/93)** | | Estrogens  Androgens  SHGB  Insulin  Insulin sensitivity | Estradiol  Oestrone  Oestrone-S  Testosterone  Androstenedione  SHBG | Weight  BMI  Waist circumference  Body fat  % body fat | - | Side effects (un-specified) |
| Orsatti FL  et al.[^44^](#_ENREF_44)  2008  Brazil | | **50** | Post-menopausal  Age: 45-70 years  Sedentary  No hormonal treatment | Age: 58.5 ± 7.1 years  BMI: 28.2 ± 4.8 kg/m^2^ | Resistance  Supervised  **(n = 21/27)** | 50-60 min  3 d/wk. | 60-80 %  1-RM | 16 weeks | **-** | | Maintain usual activities  **(n = 22/23)** | | Muscle mass  Hormonal response | Estradiol  Testosterone | % body fat | - | - |
| Wu J  et al.[^45^](#_ENREF_45)  2006  Japan | | **136** | Post-menopausal  Age: 45–60 years  Sedentary  No hormonal treatment | Age: 54.6 ± 2.9 years  BMI: 21.6 ± 2.4 kg/m^2^ | Walking  Supervised | 45 min  3 d/wk. | - | 12 months | 1/ Isoflavones  **(n = 31/34)**  2/ Placebo  **(n = 31/34)** | | Maintain usual activities  1/Isoflavones  **(n = 33/34)**  2/Placebo  **(n = 33/34)** | | Bone mass  Body composition  Serum lipids | Estradiol | Weight  BMI  Body fat | - | - |
| **Table S3 - Characteristics of eligible studies by hormonal group (continued)** | | | | | | | | | | | | | | | | | |
| **Study references Year**  **Country** | | **Population** | | | **Exercise Group(s) (n)** | | | | | | **Comparator Group(s)**  **(n)** | | **Outcomes** | | | | |
|  |  | **N** | **Inclusion criteria** | **Participants**  **(mean ± SD)** | **Type and modality** | **Duration Frequency** | **Intensity** | **Duration**  **of study** | **Co-intervention** | |  |  | **Study primary outcome(s)** | **Hormones of interest** | **Adiposity markers** | **Hormonal function** | **Side effects** |
| **Post-menopausal** | | | | | | | | | | | | | | | | | |
| Valkeinen H et al.[^46^](#_ENREF_46)  2005  Finland | | **26** | Age: 55-65 years  Fibromyalgia diagnosis  ± Hormonal treatment | Age: 59.5 ± 3.0 years  Weight: 67.5 ± 9.0 kg | Resistance  Supervised  **(n = 13)** | Duration: 6-7 exercises for all the main muscle groups.  2 d/wk. | 40-80 %  1-RM | 21 weeks | - | | Maintain usual activities  **(n = 13)** | | Muscle strength  Serum hormone | Testosterone (total+free)  DHEA-S | - | - | - |
| Copeland JL et al.[^47^](#_ENREF_47)  2004  Canada | | **32** | Post-menopausal  Age >50 years  Sedentary  ± Hormonal treatment | Age: 55.1 ± 5.7 years  BMI: 28.5 ± 5.8 kg/m^2^ | Resistance  Supervised  **(n = 16)** | Duration:  2-3 sets of 10 repetitions  of each of eight exercises  3 d/wk. | 10 RM | 12 weeks | - | | Flexibility exercises  3 d/wk.  Not supervised  **(n = 16)** | | Hormonal response | Estradiol  Testosterone  DHEA | Weight  BMI  % body fat | - | - |
| McTiernan A et al.[^48-52^](#_ENREF_48)  2004  (PATH trial)  USA | | **173** | Post-menopausal  Age: 50-75 years  BMI ≥25.0 kg/m^2^  Sedentary  No hormonal treatment | Age: 60.7 ± 6.8 years  BMI: 30.5 ± 3.9 kg/m^2^ | Aerobic  3 supervised sessions  2 sessions at home  **(n = 84/87)** | 45 min  5 d/wk. | 40 %  Gradual ↑  60-75 % HR max | 12 months | **-** | | 45 min/wk. stretching  Maintain usual activities  **(n = 85/86)** | | Hormonal and metabolic profile  Adiposity | Oestrone  Estradiol  Testosterone  DHEA  DHEA-S  Androstenedione  SHBG  2-OHE1  16α-OHE1 | Weight  BMI  Waist  circumference  Body fat  % body fat | Menopausal symptoms | Bone density |
| Figueroa A  et al.[^53^](#_ENREF_53)  2003  USA | | **94** | Post-menopausal  Age: 40-65 years  ± Hormonal treatment | Age: 56.2 ± 4.4 years  Weight: 69.0 ± 11.8 kg | Resistance and weight bearing aerobic exercise  Supervised  **(n = 36/44)** | 60-75 min  3 d/wk. | 70-80 %  1-RM  50-80 % HR max | 12 months | **-** | | Maintain usual activities  **(n = 48/50)** | | Adiposity | Oestrone  Estradiol  Androstenedione | Body fat  % body fat | - | - |
| **Polycystic ovary syndrome** | | | | | | | | | | | | | | | | | |
| Nybacka A  et al.[^54^](#_ENREF_54)^,^[^55^](#_ENREF_55)  2013  Sweden | | **57** | Age: 18-40 years  BMI >27 kg/m^2^  PCOS diagnosis  No hormonal  treatment | Age: 30.8 ± 5.2 years  BMI: 35.9 ± 6.2 kg/m^2^ | Individualised  Aerobics and strength training  Supervised | 45-60 min  2-3 d/wk. | Moderate intensity | 4 months | 1/ No diet **(n =17/19)**  2/ Diet  (**n = 12/19)** | | Diet  **(n = 14/19)** | | Ovarian function  Metabolic parameters | Testosterone  SHBG | BMI  % body fat | Menstrual regularity  Ovulation | - |
| Curi DD  et al.[^56^](#_ENREF_56)  2012  Brazil | | **40** | PCOS diagnosis  Age: 18-34 years  BMI >25 kg/m^2^  No OC | Age (years):  24.6 ± 1.3 *vs*  26.3 ± 1.4  BMI (kg/m^2^):  31.1 ±1.5 *vs*  31.8 ±1.6 | Walking +  resistance  **(n = 12/20)** | ≥40 min  Frequency:  NR | - | 6 months | Diet  (500 kcal/d restriction) | | Metformin  **(n = 15/20)** | | Menstrual cycle Hormonal and metabolic profile | Testosterone  Androstenedione  SHBG | BMI  Waist circumference | Menstrual frequency | - |
| Palomba S  et al.[^58^](#_ENREF_58)  2010  Italy | | **96** | PCOS resistant to CC  Age: 18-35 years  BMI: 25-35 kg/m^2^  No medication | Age: 27.5 ± 5.8 years  BMI: 31.5 ± 3.1 kg/m^2^ | Structured cycloergometer exercise  Supervised | 30 min  3 d/wk. | 60-70 % VO max | 6 weeks | 1/ Diet  (1000 kcal/d  restriction)  **(n = 32/32)**  2/ CC + diet  (1000 kcal/d restriction)  **(n = 32/32)** | | 1/ CC after the first 2 weeks  **(n = 32/32)** | | Ovulation | Estradiol  Testosterone  Androstenedione  DHEA-S  SHBG | Weight  BMI  Waist  circumference | Ovulation | Side effects (un-specified) |
| **Table S3 - Characteristics of eligible studies by hormonal group (continued)** | | | | | | | | | | | | | | | | | |
| **Study references**  **Year**  **Country** | | **Population** | | | **Exercise Group(s) (n)** | | | | | | **Comparator Group(s)**  **(n)** | | **Outcomes** | | | | |
|  |  | **N** | **Inclusion criteria** | **Participants**  **(mean ± SD)** | **Type and modality** | **Duration Frequency** | **Intensity** | **Duration**  **of study** | **Co-intervention** | |  |  | **Study primary outcome(s)** | **Hormones of interest** | **Adiposity markers** | **Hormonal function** | **Side effects** |
| **Polycystic ovary syndrome** | | | | | | | | | | | | | | | | | |
| Karimzadeh MA  et al.[^59^](#_ENREF_59)  2010  Iran | | **343** | Age: 19-35 years  BMI: 25–29.9 kg/m^2^  PCOS with infertility | Age: 27.4 ± 3.4 years  BMI: 27.5 ± 1.7 kg/m^2^ | Exercise  advice  (aerobic, walking, resistance)  **(n = 75)** | 20-60 min  3-5 d/wk. | Adjusted HR | 6 months | Diet advice  (500kcal/d restriction) | | 1/ Metformin  **(n = 90)**  2/ CC **(n = 90)**  3/ Metformin + CC **(n = 88)** | | Menstrual cycles  Waist  circumference  Endocrine parameters  Lipid profile | Testosterone  SHBG | BMI  Waist  circumference | Menstrual cycles  Ovulation  Clinical pregnancy | - |
| Brown AJ et al.[^60^](#_ENREF_60)  2009  USA | | **37** | Pre-menopausal  Age: 18-50 years  PCOS diagnosis  No hormonal treatment | Age: median (IQ)  36.5 (5.0) *vs*  28.0 (11.0)  BMI: median (IQ)  37.9 (9.4) *vs*  31.3 (14.9) | Aerobic  Personalised  Monitored  +/- supervised  (**n = 8**) | 60 min/d  ≈ 228 min/wk. | Moderate  14 kcal/kg/wk. | 20-24 weeks | **-** | | No exercise  **(n = 12)** | | Lipoproteins  Insulin sensibility | Bioavailable testosterone | Weight  BMI  Waist circumference | - | Side effects (un-specified) |
| Hoeger K et al.[^61^](#_ENREF_61)  2008  USA | | **43** | Post-menarchal adolescent  Age: 12-18 years  BMI >95^ème^ percentile  PCOS diagnosis  No hormonal treatment | Age: 15.5 ± 1.5 years  BMI: 36.5 ± 6.8 kg/m^2^ | Training classes for exercise  No monitored exercise  **(n = 8/11)** |  | - | 24  weeks | Training classes for diet and behavior modification | | Standard office advice on nutrition and exercise  1/ Metformin  **(n = 6/10)**  2/ Oral contraceptives  **(n = 10/11)**  3/ Placebo  **(n = 10/11)** | | Endocrine and metabolic response | Testosterone  SHBG | BMI  Waist circumference | Menstrual  cycles  Ovulation | - |
| Thomson RL et al.[^62^](#_ENREF_62)^,^[^63^](#_ENREF_63)  2008  Australia | **104** | | Age: 18-40 years  PCOS diagnosis  Overweight and obese  No OC or hormonal treatment | Age: 29.3 ± 6.8 years  BMI: 36.1 ± 4.8 kg/m2 | 1/ Aerobic:  Walking/  jogging  (**n = 18/31**)  2/ Aerobic + resistance  (**n = 20/33**) | 1/ 45 min  5 d/wk.  2/ 45 min  5 d/wk. +  2 d/wk. resistance exercise | 75-80 % HR max  75-80 % HR max | 20 weeks | 1/ High-protein diet  (1200–1500 kcal/d)  2/ High-protein diet (1200–1500 kcal/d) | | | High-protein diet (1200–1500 kcal/d)  (**n = 14/30**) | Adiposity  Cardio-metabolic and hormonal profile | Testosterone  SHBG | Weight  Body fat  % body fat Waist circumference | Menstrual regularity  Ovulation | - |
| Vigorito C  et al.[^64^](#_ENREF_64)  2007  Italy | | **90** | PCOS diagnosis  Overweight  No OC or hormonal treatment | Age: 21.8 ± 2.1 years  BMI: 29.4 ± 3.2 kg/m^2^ | Structured cycloergometer exercise training Supervised  **(n = 45)** | 30 min  3 d/wk. | 60-70 %  VO^2^ max | 3 months | **-** | | No exercise  **(n = 45)** | | Cardio-pulmonary function | Estradiol  Testosterone  Androstenedione  DHEA-S  SHBG | BMI  Waist  circumference | Normal cycles | Side effects (un-specified) |
| Bruner B  et al.[^65^](#_ENREF_65)  2006  Canada | | **12** | PCOS diagnosis  BMI >27 kg/m^2^  No OC | Age: 30.7 ± 4.0 years  BMI: 36.6 ± 6.2 kg/m^2^ | Endurance and resistance training  Supervised  **(n = 7/7)** | 90 min  3 d/wk. | 70-85 % HR max | 12 weeks | Nutritional  counseling  (group nutritional seminars) | | Nutritional  counseling  (group nutritional seminars)  **(n = 4/5)** | | Metabolic and reproductive function | Testosterone  SHBG | Weight  BMI  Waist  circumference | Normal cycles | - |
| Hoeger KM et al.[^66^](#_ENREF_66)  2004  USA | | **38** | PCOS diagnosis  BMI >25 kg/m^2^.  No hormonal treatment | Age: 28.4 ± 5.1 years  BMI: 39.0 ± 5.8 kg/m^2^ | Individualized exercise programs recommendations | 150 min/wk. | - | 48 weeks | Diet plan  (restriction of 500–1000 Cal/d)  1/ Metformin  **(n = 5/9)**  2/ Placebo  **(n = 6/11)** | | 1/Metformin  **(n = 5/9)**  2/Placebo  **(n = 7/ 9)** | | Compliance | Testosterone  SHBG | Weight | Ovulation  Menstrual  events | Metfor-min side effects |
| **Table S3 - Characteristics of eligible studies by hormonal group (continued)** | | | | | | | | | | | | | | | | | |
| **Study**  **references**  **Year**  **Country** | | **Population** | | | **Exercise Group(s) (n)** | | | | | | **Comparator Group(s)**  **(n)** | | **Outcomes** | | | | |
|  |  | **N** | **Inclusion criteria** | **Participants**  **(mean ± SD)** | **Type and modality** | **Duration Frequency** | **Intensity** | **Duration**  **of study** | **Co-intervention** | |  |  | **Study primary outcome(s)** | **Hormones of interest** | **Adiposity markers** | **Hormonal function** | **Side effects** |
| **Polycystic ovary syndrome** | | | | | | | | | | | | | | | | | |
| Guzick DS  et al.[^67^](#_ENREF_67)  1994  USA | | **12** | Age: 20-40 years  130-200% of ideal body weight  PCOS diagnosis | Age: 32.0 ± 4.4 years  Weight: 108.5 ± 5.3 kg | Walking  **(n = 6)** | 2 miles/d  5 d/wk. | - | 12 weeks | Very low calorie diet | | No exercise  **(n = 6)** | | Ovulation  Hormonal and metabolic profile | Testosterone  (total + free)  SHBG | Weight | Ovulation | Physical or psycho-logical difficul-ties |
| **Unspecified hormonal Status** | | | | | | | | | | | | | | | | | |
| von Thiele Schwarz U  et al.[^68^](#_ENREF_68)  2008  Sweden | | **177** | Female employees from six workplaces in a large public dental health care organization | Age: 46.6 ± 10.7 years  BMI: NR | Free choice  During  work-hours  **(n = 58/62)** | 1-2.5 h/wk. | 55-89 % HR max | 12 months |  | | 1/ Reduced-work-hours  **(n = 37/50)**  2/ No intervention  **(n = 61/65)** | | Lipids  Cardio- metabolic factors  Neuro-endocrine markers  Self-ratings of health, symptoms, and work ability | DHEA-S | - | - | - |
| **Per-partum** | | | | | | | | | | | | | | | | | |
| Seneviratne SN et al.[^20^](#_ENREF_20)  2014  New-Zealand | | **100** | Pregnancy: <20 wk.  Age: 18-40 years  BMI ≥25 kg/m^2^ | -  (Ongoing study) | Home-based stationary  cycling  Monitored  Distant supervision | 15-30 min  3-5 d/wk. | Moderate | 16 weeks | - | | Maintain usual activities | | Offspring birth weight | SHBG | Weight  Body composition | - | - |
| Field T  et al.[^69^](#_ENREF_69)  2013 [88]  USA | | **79** | Age: 20-40 years  Pregnancy  Depression | Age: 24.9 ± 5.2 years  BMI: NR | Tai chi/yoga  Supervised  Group sessions  **(n = 40/46)** | 20 min  1 d/wk. | - | 12 weeks | - | | Social support (group)  **(n = 39/46)** | | Depression  Anxiety  Cortisol | Estriol | - | - | - |
| **Post-partum** | | | | | | | | | | | | | | | | | |
| Zourladani A et al.[^70^](#_ENREF_70)  2014  Greece | | 42 | Healthy  Primiparous  4-6 weeks post-partum  Lactating | Age: 31.3 ± 2.5 years  Weight: 65.4 ± 10.0 kg | Aerobic+  resistance  Supervised  (**n = 20/22**) | 50-60 min  3 d/wk. | 70 %  HR max | 12 weeks | - | | No exercise  (**n = 17/20**) | | Physical  fitness  Metabolic and  hormonal profile | Estradiol | Weight  Body fat | - | - |
| Colleran HL et al.[^71^](#_ENREF_71)  2012  USA | | **31** | <3 weeks post-partum  Age: 23-37 years  BMI: 25-30 kg/m^2^  Fully breast-feeding  Sedentary | Age: 31.1 ± 3.4 years  BMI: 28.9 ± 3.4 kg/m^2^  Postpartum:  4.3 ± 0.8 weeks | Walking/ aerobic  + Resistance  Supervised  **(n = 14)** | 5 d/wk.  3 d/wk. | 10000 walking steps  or 3000 aerobic steps | 16 weeks | 500 kcal restricted diet | | Minimal care (standard  public health information)  **(n = 11/12)** | | Bone mineral density | Estradiol | Weight  Waist circumference | - | Bone mineral density  Infant growth |
| N = number of randomized participants; n = number of participants with available estrogen measures; BMI = Body Mass Index; SD = Standard Deviation; OC = Oral Contraceptives; dg = diagnosis; lbs = pounds  AUC = Area Under the Curve; EMC = Estrogen Metabolite Concentration (2-OHE1 and 16a-OHE1); DHEA = Dehydroepiandesterone; DHEAS = dehydroepiandesterone sulfate; Oestrone-S = Oestrone sulfate; SHBP = Sex Hormone Binding Protein; PCOS = Polycystic Ovary Syndrome; CC = Clominophene Citrate; HR = Heart Rate; HR max= Heart Rate max; 1-RM = Repetitions Maximum (the greatest weight that can be moved once in good form); Supervised = interaction with a coach; Monitored = monitoring device; NR = Not Reported; | | | | | | | | | | | | | | | | | |

| **Table S4 – Subgroup analyses of primary outcomes** | | | | | | | | | | |
| --- | --- | --- | --- | --- | --- | --- | --- | --- | --- | --- |
| **Subgroups** | **Total estradiol**  (Standardized Mean Difference) | | | | **Free estradiol**  (Standardized Mean Difference) | | | | | |
|  | Studies (n) | Participants (n) | Effect estimate [95% CI ] | I^2^ % | Studies (n) | Participants (n) | Effect estimate [95% CI] | I^2^ % | | |
| **Hormonal group** | | | | | | | | | | |
| Premenopausal | 4 | 495 | -0.04 [-0.22, 0.13] | 0 | 1 | 319 | -0.30 [-0.52, -0.08] | | | NA |
| Peri-menopausal | 1 | 28 | 0.01 [-0.77, 0.78] | NA | 1 | 28 | 0.05 [-0.72, 0.83] | | | NA |
| Post-menopausal | 8 | 1148 | -0.16 [-0.32, 0.00] | 29 | 3 | 898 | -0.17 [-0.30, -0.04] | | | 0 |
| Polycystic Ovary Syndrome (PCOS) | 3 | 260 | -0.19 [-0.44, 0.06] | 0 | 0 | - | - | | | - |
| Post-partum | 2 | 63 | 0.19 [-0.30, 0.69] | 0 | 0 | - | - | | | - |
| **BMI at baseline** | | | | | | | | | | |
| <25 kg/m^2^ | 5 | 603 | -0.02 [-0.18, 0.14] | 0 | 1 | 319 | 0.30 [-0.52, -0.08] | | | NA |
| 25-30 kg/m^2^ | 9 | 668 | -0.26 [-0.41, -0.11] | 0 | 2 | 337 | -0.27 [-0.48, -0.05] | | | 0 |
| ≥ 30 kg/m^2^ | 3 | 686 | -0.08 [-0.23, 0.07] | 0 | 2 | 589 | -0.11 [-0.27, 0.05] | | | 0 |
| Unknown | 1 | 37 | 0.26 [-0.39, 0.91] | NA | 0 | - | - | | | - |
| **Co-intervention group** | | | | | | | | | | |
| No co-intervention | 14 | 1408 | -0.15 [-0.25, -0.04] | 0 | 5 | 1245 | -0.20 [-0.31, -0.09] | | | 0 |
| Diet co-intervention | 3 | 353 | -0.10 [-0.31, 0.12] | 0 | 0 | - | - | | | - |
| Other co-intervention | 4 | 233 | 0.01 [-0.25, 0.27] | 0 | 0 | - | - | | | - |
| **Comparator group** | | | | | | | | | | |
| No intervention comparator | 12 | 1159 | -0.14 [-0.27, -0.02] | 5 | 3 | 656 | -0.28 [-0.44, -0.13] | | | 0 |
| Diet comparator | 1 | 231 | -0.08 [-0.33, 0.18] | NA | 1 | 420 | -0.13 [-0.32, 0.06] | | | NA |
| Other comparator | 8 | 604 | -0.09 [-0.25, 0.07] | 0 | 1 | 169 | -0.06 [-0.36, 0.25] | | | NA |
| **Duration of the intervention** | | | | | | | | | | |
| <3 months | 1 | 96 | -0.21 [-0.64, 0.21] | NA | 0 | - | - | | | - |
| 3-6 months | 8 | 598 | -0.07 [-0.23, 0.09] | 0 | 1 | 319 | -0.30 [-0.52, -0.08] | | | NA |
| 6-12 months | 4 | 273 | -0.06 [-0.30, 0.19] | 0 | 1 | 28 | 0.05 [-0.72, 0.83] | | | NA |
| ≥12 months | 5 | 1027 | -0.14 [-0.32, 0.04] | 44 | 3 | 898 | -0.17 [-0.30, -0.04] | | | 0 |
| **Type of exercise** | | | | | | | | | | |
| Endurance or aerobic exercise | 11 | 1681 | -0.14 [-0.23, -0.04] | 0 | 4 | 1217 | -0.20 [-0.32, -0.09] | | | 0 |
| Resistance or muscle-strengthening exercise | 2 | 75 | -0.41 [-0.87, 0.05] | 0 | 0 | - | - | | | - |
| Endurance and resistance exercise | 4 | 112 | 0.16 [-0.21, 0.54] | 0 | 1 | 28 | 0.05 [-0.72, 0.83] | | | NA |
| Yoga | 1 | 126 | 0.08 [-0.27, 0.42] | NA | 0 | - | - | | | - |
| **Exercise intensity** | | | | | | | | | | |
| Light | 1 | 21 | 0.28 [-0.58, 1.14] | NA | 0 | - | - | | | - |
| Moderate | 5 | 345 | -0.07 [-0.29, 0.14] | 0 | 1 | 169 | -0.06 [-0.36, 0.25] | | | NA |
| Hard | 8 | 1338 | -0.17 [-0.30, -0.05] | 15 | 4 | 1076 | -0.22 [-0.34, -0.10] | | | 0 |
| Unknown | 4 | 290 | 0.04 [-0.19, 0.27] | 0 | 0 | - | - | | | - |
| **Exercise frequency** | | | | | | | | | | |
| 3-5 days/week | 11 | 596 | -0.11 [-0.28, 0.05] | 0 | 0 | - | - | | - | |
| ≥5 days/week | 7 | 1398 | -0.11 [-0.24, 0.02] | 28 | 5 | 1245 | -0.20 [-0.31, -0.09] | | 0 | |
| **Hours of exercise per week** | | | | | | | | | | |
| <3 h/week | 10 | 829 | -0.08 [-0.21, 0.06] | 0 | 1 | 319 | -0.30 [-0.52, -0.08] | | NA | |
| 3-5 h/week | 6 | 1105 | -0.13 [-0.33, 0.06] | 54 | 3 | 898 | -0.17 [-0.30, -0.04] | | 0 | |
| ≥5 h/week | 1 | 28 | 0.01 [-0.77, 0.78] | NA | 1 | 28 | 0.05 [-0.72, 0.83] | | NA | |
| Unknown | 1 | 32 | -0.21 [-0.90, 0.49] | NA | 0 | - | - | | - | |
| **Exercise modality: supervision/monitoring** | | | | | | | | | | |
| Direct or indirect supervision ± monitored exercise | 13 | 1020 | -0.07 [-0.19, 0.06] | 0 | 2 | 347 | -0.27 [-0.49, -0.06] | | 0 | |
| Monitored only exercise | 1 | 45 | 0.00 [-0.60, 0.60] | NA | 0 | - | - | | - | |
| Mix of supervised and not supervised exercise | 3 | 899 | -0.17 [-0.40, 0.06] | 65 | 3 | 898 | -0.17 [-0.30, -0.04] | | 0 | |
| Unknown | 1 | 30 | -0.23 [-0.95, 0.49] | NA | 0 | - | - | | - | |
| **Exercise modality: group sessions** | | | | | | | | | | |
| Exercise within group sessions | 2 | 730 | -0.23 [-0.55, 0.09] | 79 | 2 | 729 | -0.20 [-0.36, -0.04] | | 16 | |
| Unknown | 16 | 1264 | -0.06 [-0.17, 0.05] | 0 | 3 | 516 | -0.20 [-0.38, -0.02] | | 5 | |
| **Weight loss after intervention** | | | | | | | | | | |
| Significant weight loss | 6 | 1043 | -0.14 [-0.30, 0.02] | 26 | 4 | 926 | -0.16 [-0.29, -0.04] | | 0 | |
| No weight loss | 9 | 775 | -0.09 [-0.23, 0.05] | 0 | 1 | 319 | -0.30 [-0.52, -0.08] | | NA | |
| Unknown | 3 | 176 | -0.01 [-0.31, 0.28] | 0 | 0 | - | - | | - | |
| NA = Not applicable; CI = Confidence interval | | | | | | | | | | |

| **Table S5 – Subgroup analyses of secondary outcomes : Total and free testosterone** | | | | | | | | | | | | | | | | | | | | | | | |
| --- | --- | --- | --- | --- | --- | --- | --- | --- | --- | --- | --- | --- | --- | --- | --- | --- | --- | --- | --- | --- | --- | --- | --- |
| **Subgroups** | | | | | | **Total testosterone**  (Standardized Mean Difference) | | | | | | | | | **Free testosterone**  (Mean Difference, pg/ml) | | | | | | | | |
|  |  |  |  |  |  | Studies (n) | Participants (n) | | | | Effect estimate [95% CI] | | | I^2^ % | Studies (n) | | | Participants (n) | | Effect estimate [95% CI] | | I^2^ % | |
| **Hormonal group** | | | | | | | | | | | | | | | | | | | | | | | |
| Premenopausal | | | | | | 5 | 502 | | | | -0.07 [-0.37, 0.23] | | | 41 | 3 | | | 357 | | -0.28 [-0.65, 0.09] | | 28 | |
| Peri-menopausal | | | | | | 1 | 28 | | | | -0.18 [-0.96, 0.59] | | | NA | 0 | | | - | | - | | - | |
| Post-menopausal | | | | | | 7 | 1022 | | | | -0.17 [-0.38, 0.05] | | | 48 | 4 | | | 926 | | -0.24 [-0.44, -0.04] | | 0 | |
| Polycystic Ovary Syndrome (PCOS) | | | | | | 8 | 387 | | | | 0.00 [-0.41, 0.41] | | | 69 | 2 | | | 86 | | -2.26 [-17.83, 13.32] | | 19 | |
| Post-partum | | | | | | 0 | - | | | | - | | | - | 0 | | | - | | - | | - | |
| **BMI at baseline** | | | | | | | | | | | | | | | | | | | | | | | |
| <25 kg/m^2^ | | | | | | 4 | 481 | | | | -0.03 [-0.37, 0.31] | | | 51 | 2 | | | 336 | | -0.29 [-0.94, 0.35] | | 35 | |
| 25-30 kg/m^2^ | | | | | | 7 | 597 | | | | -0.25 [-0.57, 0.06] | | | 61 | 2 | | | 383 | | -0.20 [-0.51, 0.11] | | 0 | |
| ≥30 kg/m^2^ | | | | | | 7 | 802 | | | | 0.07 [-0.23, 0.36] | | | 62 | 2 | | | 591 | | -0.09 [-0.49, 0.32] | | 0 | |
| Unknown | | | | | | 3 | 59 | | | | -0.59 [-1.11, -0.06] | | | 0 | 3 | | | 59 | | -0.37 [-0.66, -0.08] | | 0 | |
| **Co-intervention group** | | | | | | | | | | | | | | | | | | | | | | | |
| No co-intervention | | | | | | 17 | 1555 | | | | -0.10 [-0.28, 0.09] | | | 59 | 8 | | | 1357 | | -0.18 [-0.29, -0.07] | | 0 | |
| Diet co-intervention | | | | | | 4 | 350 | | | | -0.23 [-0.44, -0.02] | | | 0 | 1 | | | 12 | | -27.00 [-75.26, 21.26] | | NA | |
| Other co-intervention | | | | | | 1 | 34 | | | | 0.36 [-0.44, 1.16] | | | NA | 0 | | | - | | - | | - | |
| **Comparator groups** | | | | | | | | | | | | | | | | | | | | | | | |
| No intervention comparator | | | | | | 12 | 1204 | | | | -0.21 [-0.41, -0.00] | | | 54 | 6 | | | 1108 | | -0.28 [-0.47, -0.08] | | 0 | |
| Diet comparator | | | | | | 3 | 285 | | | | -0.09 [-0.32, 0.15] | | | 0 | 0 | | | - | | - | | - | |
| Other comparator | | | | | | 7 | 450 | | | | 0.06 [-0.31, 0.43] | | | 66 | 3 | | | 261 | | -0.14 [-0.27, -0.01] | | 0 | |
| **Duration of the intervention** | | | | | | | | | | | | | | | | | | | | | | | |
| <3 months | | | | | | 2 | 113 | | | | -0.47 [-0.86, -0.07] | | | 0 | 1 | | | 17 | | -0.14 [-0.27, -0.01] | | NA | |
| 3-6 months | | | | | | 11 | 637 | | | | -0.22 [-0.53, 0.09] | | | 59 | 4 | | | 378 | | -0.40 [-0.68, -0.11] | | 0 | |
| 6-12 months | | | | | | 5 | 289 | | | | 0.22 [-0.22, 0.65] | | | 62 | 1 | | | 74 | | 0.30 [-0.89, 1.48] | | NA | |
| ≥12 months | | | | | | 3 | 900 | | | | -0.07 [-0.21, 0.06] | | | 0 | 3 | | | 900 | | -0.18 [-0.43, 0.07] | | 0 | |
| **Type of exercise** | | | | | | | | | | | | | | | | | | | | | | | |
| Endurance or aerobic exercise | | | | | | 8 | 1491 | | | | -0.15 [-0.26, -0.03] | | | 16 | 6 | | | 1305 | | -0.19 [-0.43, 0.05] | | 0 | |
| Resistance or muscle-strengthening exercise | | | | | | 5 | 132 | | | | -0.05 [-1.06, 0.96] | | | 86 | 2 | | | 43 | | -0.18 [-0.33, -0.03] | | 17 | |
| Endurance and resistance exercise | | | | | | 6 | 156 | | | | 0.04 [-0.28, 0.37] | | | 0 | 1 | | | 21 | | -0.57 [-1.30, 0.16] | | NA | |
| Yoga | | | | | | 1 | 126 | | | | -0.02 [-0.37, 0.32] | | | NA | 0 | | | - | | - | | - | |
| Unknown | | | | | | 1 | 34 | | | | 0.36 [-0.44, 1.16] | | | NA | 0 | | | - | | - | | - | |
| **Exercise intensity** | | | | | | | | | | | | | | | | | | | | | | | |
| Light | | | | | | 1 | 21 | | | | 0.00 [-0.86, 0.86] | | | NA | 0 | | | - | | - | | - | |
| Moderate | | | | | | 3 | 287 | | | | -0.05 [-0.28, 0.19] | | | 0 | 2 | | | 244 | | -0.14 [-0.72, 0.44] | | 0 | |
| Hard | | | | | | 12 | 1415 | | | | -0.22 [-0.41, -0.02] | | | 56 | 5 | | | 1096 | | -0.28 [-0.47, -0.08] | | 0 | |
| Unknown | | | | | | 5 | 216 | | | | 0.10 [-0.55, 0.75] | | | 72 | 2 | | | 29 | | -0.14 [-0.27, -0.01] | | 16 | |
| **Exercise frequency** | | | | | | | | | | | | | | | | | | | | | | | |
| <3 days/week | | | | | | 4 | 107 | | | | -0.30 [-0.76, 0.15] | | | 24 | 3 | | | 64 | | -0.20 [-0.36, -0.04] | | 14 | |
| 3-5 days/week | | | | | | 8 | 386 | | | | -0.19 [-0.57, 0.19] | | | 66 | 1 | | | 74 | | 0.30 [-0.89, 1.48] | | NA | |
| ≥5 days/week | | | | | | 7 | 1385 | | | | -0.09 [-0.19, 0.02] | | | 0 | 5 | | | 1231 | | -0.21 [-0.46, 0.04] | | 0 | |
| Unknown | | | | | | 2 | 61 | | | | 0.87 [-0.14, 1.87] | | | 66 | 0 | | | - | | - | | - | |
| **Hours of exercise per week** | | | | | | | | | | | | | | | | | | | | | | | |
| <3 h/week | | | | | | 5 | 600 | | | | -0.24 [-0.45, -0.02] | | | 28 | 2 | | | 393 | | -0.31 [-1.58, 0.96] | | 50 | |
| 3-5 h/week | | | | | | 8 | 1142 | | | | -0.08 [-0.30, 0.13] | | | 57 | 3 | | | 900 | | -0.18 [-0.43, 0.07] | | 0 | |
| ≥5 h/week | | | | | | 1 | 28 | | | | -0.18 [-0.96, 0.59] | | | NA | 0 | | | - | | - | | - | |
| Unknown | | | | | | 7 | 169 | | | | -0.03 [-0.61, 0.55] | | | 68 | 4 | | | 76 | | -0.21 [-0.38, -0.04] | | 15 | |
| **Exercise modality: supervision/monitoring** | | | | | | | | | | | | | | | | | | | | | | | |
| Direct or indirect supervision ± monitored exercise | | | | | | 15 | 966 | | | | -0.20 [-0.41, 0.00] | | | 48 | 5 | | | 457 | | -0.20 [-0.36, -0.05] | | 8 | |
| Monitored-only exercise | | | | | | 0 | - | | | | - | | | - | 0 | | | - | | - | | - | |
| Mix of supervised and not supervised exercise | | | | | | 3 | 900 | | | | -0.07 [-0.21, 0.06] | | | 0 | 3 | | | 900 | | -0.18 [-0.43, 0.07] | | 0 | |
| Not supervised, not monitored | | | | | | 2 | 46 | | | | -0.12 [-1.24, 0.99] | | | 60 | 1 | | | 12 | | -27.00 [-75.26, 21.26] | | NA | |
| Unknown | | | | | | 1 | 27 | | | | 1.39 [0.53, 2.25] | | | NA | 0 | | | - | | - | | - | |
| **Exercise modality: group sessions** | | | | | | | | | | | | | | | | | | | | | | | |
| Exercise within group sessions | | | | | | 2 | 730 | | | | -0.07 [-0.21, 0.08] | | | 0 | 2 | | | 730 | | -0.17 [-0.44, 0.10] | | 0 | |
| Unknown | | | | | | 19 | 1209 | | | | -0.12 [-0.33, 0.08] | | | 57 | 7 | | | 639 | | -0.18 [-0.30, -0.07] | | 0 | |
| **Weight loss after intervention** | | | | | | | | | | | | | | | | | | | | | | | |
| Significant weight loss | | | | | | 8 | 1100 | | | | -0.07 [-0.31, 0.17] | | | 63 | 4 | | | 912 | | -0.18 [-0.43, 0.07] | | 0 | |
| No weight loss | | | | | | 10 | 670 | | | | -0.11 [-0.40, 0.18] | | | 55 | 3 | | | 414 | | -0.44 [-1.06, 0.18] | | 11 | |
| Unknown | | | | | | 3 | 169 | | | | -0.29 [-0.76, 0.17] | | | 35 | 2 | | | 43 | | -0.18 [-0.33, -0.03] | | 17 | |
| NA = Not Applicable ; CI = Confidence Interval | | | | | |  |  | | | |  | | |  |  | | |  | |  | |  | |
| **Table S6– Subgroup analyses of secondary outcomes : Androstenedione, DHEA-sulfate, SHBG** | | | | | | | | | | | | | | | | | | | | | | | |
| **Subgroups** | **Androstenedione**  (Mean Difference, pg/ml) | | | | | | | | | **DHEA-sulfate**  (Mean Difference, μmol/l) | | | | | | | **SHBG**  (Mean Difference, nmol/l) | | | | | | |
|  | Studies (n) | | Participants (n) | Effect estimate [95%CI] | | | | I^2^ % | | Studies (n) | | Participants (n) | Effect estimate [95%CI] | | | I^2^ % | Studies (n) | | Participants (n) | | Effect estimate [95%CI] | | I^2^ % |
| **Hormonal group** |  | |  |  | | | |  | | | | | | | | | | | | | | | |
| Premenopausal | 0 | | - | - | | | | - | | 2 | | 147 | -0.25 [-0.97, 0.47] | | | 0 | 2 | | 352 | | 2.14 [-1.45, 5.73] | | 0 |
| Peri-menopausal | 0 | | - | - | | | | - | | 1 | | 28 | -0.87 [-2.58, 0.84] | | | NA | 0 | | - | | - | | - |
| Post-menopausal | 5 | | 900 | -25.84 [-57.16, 5.49] | | | | 0 | | 2 | | 196 | -0.44 [-0.78, -0.09] | | | 0 | 4 | | 929 | | 1.27 [-1.11, 3.65] | | 0 |
| PCOS | 4 | | 287 | -50.61 [-134.59, 33.37] | | | | 29 | | 2 | | 170 | -0.30 [-0.89, 0.29] | | | 0 | 8 | | 353 | | 6.76 [5.56, 7.96] | | 77 |
| Not specified | 0 | | - | - | | | | - | | 1 | | 156 | 0.19 [-0.49, 0.87] | | | NA | 0 | | - | | - | | - |
| **BMI at baseline** | | | | | | | | | | | | | | | | | | | | | | | |
| <25 kg/m^2^ | 0 | |  |  | | | |  | | 1 | | 126 | -0.22 [-1.15, 0.71] | | | NA | 2 | | 352 | | 2.14 [-1.45, 5.73] | | 0 |
| 25- 0 kg/m^2^ | 3 | | 473 | | -37.88 [-89.68, 13.92] | | | 0 | | 2 | | 102 | -0.33 [-0.99, 0.33] | | | 0 | 4 | | 485 | | 1.71 [-0.43, 3.85] | | 0 |
| ≥30 kg/m^2^ | 4 | | 714 | | -36.27 [-91.74, 19.20] | | | 49 | | 2 | | 266 | -0.39 [-0.76, -0.02] | | | 0 | 7 | | 785 | | 4.64 [0.12, 9.17] | | 78 |
| Unknown | 0 | | - | | - | | | - | | 3 | | 203 | -0.22 [-0.77, 0.32] | | | 25 | 1 | | 12 | | 28.89 [5.35, 52.43] | | NA |
| **Co-intervention group** | | | | | | | | | | | | | | | | | | | | | | | |
| No co-intervention | 6 | | 1091 | -26.35 [-55.54, 2.84] | | | | 0 | | 7 | | 601 | -0.31 [-0.57, -0.05] | | | 0 | 7 | | 1163 | | 2.03 [0.39, 3.67] | | 0 |
| Diet co-intervention | 1 | | 96 | -110.31 [-211.69, -8.93] | | | | NA | | 1 | | 96 | -0.44 [-1.49, 0.61] | | | NA | 8 | | 471 | | 6.64 [1.26, 12.03] | | 70 |
| **Comparator group** | | | | | | | | | | | | | | | | | | | | | | | |
| No intervention comparator | 2 | | 399 | -42.52 [-95.79, 10.75] | | | | 0 | | 3 | | 75 | -0.58 [-1.16, 0.00] | | | 0 | 6 | | 950 | | 2.87 [0.50, 5.23] | | 35 |
| Diet comparator | 1 | | 421 | -4.98 [-52.16, 42.20] | | | | NA | | 0 | | - | - | | | - | 3 | | 285 | | -1.35 [-7.18, 4.48] | | 10 |
| Other comparator | 4 | | 367 | -47.88 [-120.45, 24.68] | | | | 30 | | 4 | | 466 | -0.34 [-0.65, -0.03] | | | 0 | 6 | | 399 | | 5.97 [1.82, 10.12] | | 53 |
| **Duration of the intervention** | | | | | | | | | | | | | | | | | | | | | | | |
| <3 months | 1 | | 96 | -110.31 [-211.69, -8.93] | | | | NA | | 1 | | 96 | -0.44 [-1.49, 0.61] | | | NA | 1 | | 96 | | 8.35 [6.93, 9.77] | | NA |
| 3-6 months | 1 | | 90 | -57.30 [-146.28, 31.68] | | | | NA | | 2 | | 47 | -0.54 [-1.16, 0.08] | | | 0 | 7 | | 538 | | 2.54 [-0.65, 5.74] | | 28 |
| 6-12 months | 2 | | 101 | 93.47 [-94.78, 281.72] | | | | 0 | | 3 | | 228 | -0.30 [-0.83, 0.24] | | | 0 | 3 | | 101 | | 7.38 [2.64, 12.11] | | 0 |
| ≥12 months | 3 | | 900 | -25.83 [-57.16, 5.49] | | | | 0 | | 2 | | 326 | -0.17 [-0.71, 0.37] | | | 50 | 3 | | 899 | | 0.93 [-1.58, 3.44] | | 0 |
| **Type of exercise** | | | | | | | | | | | | | | | | | | | | | | | |
| Endurance exercise | 6 | | 1160 | -34.39 [-62.53, -6.25] | | | | 0 | | 3 | | 340 | -0.36 [-0.68, -0.03] | | | 0 | 10 | | 1535 | | 3.22 [-0.15, 6.59] | | 82 |
| Resistance exercise | 1 | | 27 | 222.00 [-132.42, 576.42] | | | | NA | | 1 | | 26 | -0.64 [-1.37, 0.09] | | | NA | 0 | | **-** | | **-** | | - |
| Endurance and Resistance exercise | 0 | | - | - | | | | - | | 2 | | 49 | -0.48 [-1.44, 0.48] | | | 0 | 3 | | 81 | | 6.79 [0.82, 12.75] | | 10 |
| Yoga | 0 | | - | - | | | | - | | 1 | | 126 | -0.22 [-1.15, 0.71] | | | NA | 0 | | - | | - | | - |
| Free |  | |  |  | | | |  | | 1 | | 156 | 0.19 [-0.49, 0.87] | | | NA | 1 | | 18 | | 12.90 [-3.23, 29.03] | | NA |
| **Exercise intensity** | | | | | | | | | | | | | | | | | | | | | | | |
| Moderate | 2 | | 244 | | -42.51 [-94.93, 9.91] | | | 0 | | 2 | | 244 | -0.35 [-0.69, -0.00] | | | 0 | 3 | | 268 | | -0.57 [-5.57, 4.42] | | 0 |
| Hard | 4 | | 916 | | -35.84 [-75.61, 3.92] | | | 22 | | 5 | | 327 | -0.28 [-0.69, 0.12] | | | 0 | 8 | | 1309 | | 3.57 [-0.02, 7.15] | | 83 |
| Unknown | 1 | | 27 | | 222.00 [-132.42, 576.42] | | | NA | | 1 | | 126 | -0.22 [-1.15, 0.71] | | | NA | 3 | | 57 | | 11.88 [2.58, 21.18] | | 35 |
| **Exercise frequency** | | | | | | | | | | | | | | | | | | | | | | | |
| <3 days/week | 0 | | - | | - | | | | - | 2 | | 47 | -0.54 [-1.16, 0.08] | | | 0 | 1 | | 43 | | -0.89 [-13.91, 12.13] | | NA |
| 3-5 days/week | 3 | | 260 | | -70.12 [-134.16, -6.08] | | | | 0 | 2 | | 170 | -0.30 [-0.89, 0.29] | | | 0 | 6 | | 316 | | 4.94 [-0.19, 10.06] | | 80 |
| ≥5 days/week | 3 | | 900 | | -25.83 [-57.16, 5.49] | | | | 0 | 3 | | 324 | -0.38 [-0.73, -0.02] | | | 0 | 5 | | 1230 | | 1.65 [-1.26, 4.55] | | 40 |
| Unknown | 1 | | 27 | | 222.00 [-132.42, 576.42] | | | | NA | 1 | | 156 | 0.19 [-0.49, 0.87] | | | NA | 2 | | 45 | | 8.49 [3.34, 13.64] | | 0 |
| **Hours of exercise per week** | | | | | | | | | | | | | | | | | | | | | | | |
| <3 h/week | 3 | | 260 | -70.12 [-134.16, -6.08] | | | | 0 | | 3 | | 326 | -0.09 [-0.54, 0.35] | | | 0 | 4 | | 561 | | 3.62 [-1.37, 8.60] | | 90 |
| 3-5 h/week | 3 | | 900 | -25.83 [-57.16, 5.49] | | | | 0 | | 2 | | 296 | -0.36 [-0.72, 0.01] | | | 0 | 6 | | 983 | | 1.32 [-1.01, 3.66] | | 0 |
| ≥5 h/week | 0 | | - | - | | | | - | | 1 | | 28 | -0.87 [-2.58, 0.84] | | | NA | 1 | | 33 | | 23.50 [-21.18, 68.18] | | NA |
| Unknown | 1 | | 27 | 222.00 [-132.42, 576.42] | | | | NA | | 2 | | 47 | -0.54 [-1.16, 0.08] | | | 0 | 3 | | 57 | | 11.88 [2.58, 21.18] | | 35 |
| **Exercise modality: supervision/monitoring** | | | | | | | | | | | | | | | | | | | | | | | |
| Direct or indirect supervision ± monitored exercise | 3 | | 260 | -70.12 [-134.16, -6.08] | | | | 0 | | 6 | | 371 | -0.41 [-0.78, -0.03] | | | 0 | 8 | | 678 | | 3.88 [-0.20, 7.96] | | 78 |
| Monitored only exercise | 0 | | - | - | | | | - | | 0 | | - | - | | | - | 0 | | - | | - | | - |
| Supervised and not supervised exercise | 3 | | 900 | -25.83 [-57.16, 5.49] | | | | 0 | | 1 | | 170 | -0.38 [-0.77, 0.01] | | | NA | 3 | | 899 | | 0.93 [-1.58, 3.44] | | 0 |
| Not supervised, not monitored | 0 | | - | - | | | | - | | 0 | | - | - | | | - | 2 | | 30 | | 18.50 [3.55, 33.45] | | 17 |
| Unknown | 1 | | 27 | 222.00 [-132.42, 576.42] | | | | NA | | 1 | | 156 | 0.19 [-0.49, 0.87] | | | NA | 1 | | 27 | | 7.99 [2.56, 13.42] | | NA |
| **Exercise modality: group sessions** | | | | | | | | | | | | | | | | | | | | | | | |
| Exercise within group sessions | 2 | | 730 | -14.79 [-53.26, 23.69] | | | | 0 | | 0 | | - | - | | | - | 3 | | 760 | | 1.67 [-0.92, 4.27] | | 0 |
| Unknown | 5 | | 457 | -53.21 [-98.09, -8.34] | | | | 7 | | 8 | | 697 | -0.31 [-0.57, -0.06] | | | 0 | 11 | | 874 | | 4.71 [1.01, 8.41] | | 76 |
| **Weight loss after intervention** | | | | | | | | | | | | | | | | | | | | | | | |
| Significant weight loss | 5 | 1017 | | -27.57 [-57.02, 1.88] | | | | 0 | | 2 | | 198 | -0.40 [-0.79, -0.02] | | | 0 | 9 | | 1134 | | 2.51 [-0.08, 5.09] | | 41 |
| No weight loss | 2 | 170 | | -66.87 [-202.25, 68.50] | | | | 34 | | 3 | | 191 | -0.30 [-0.83, 0.22] | | | 0 | 5 | | 500 | | 5.90 [1.02, 10.79] | | 68 |
| Unknown | 0 | - | | - | | | | - | | 3 | | 308 | -0.21 [-0.72, 0.30] | | | 24 | 0 | | - | | - | | - |
| PCOS = Polycystic Ovary Syndrome; NA = Not Applicable; DHEA = Dehydroepiandrosterone; SHBG = Sex Hormone Binding Globulin; | | | | | | | | | | | | | | | | | | | | | | | |


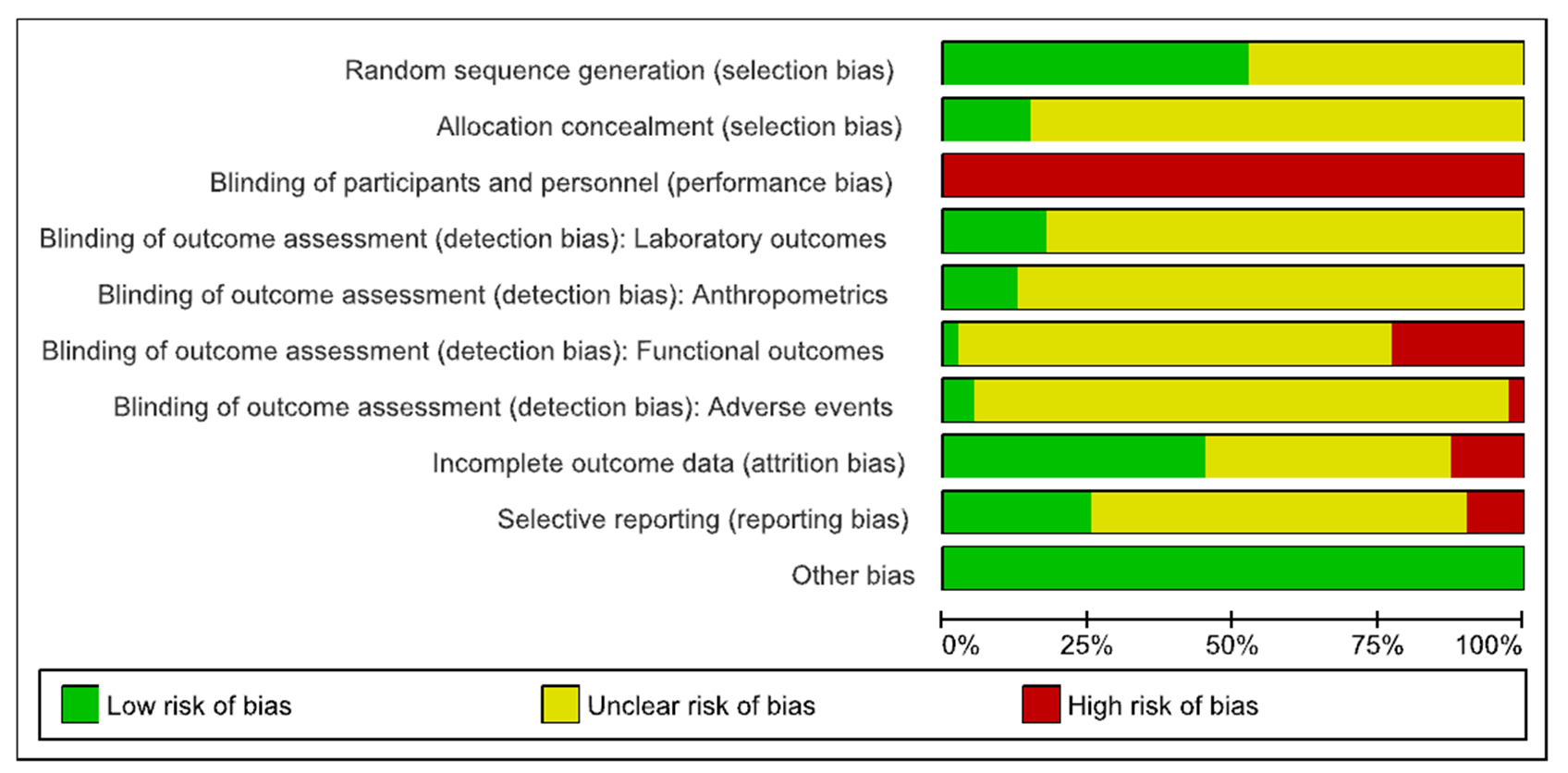


**Figure S1** **-** Risk of bias graph: review authors' judgements about each risk of bias item presented as percentages across all included studies


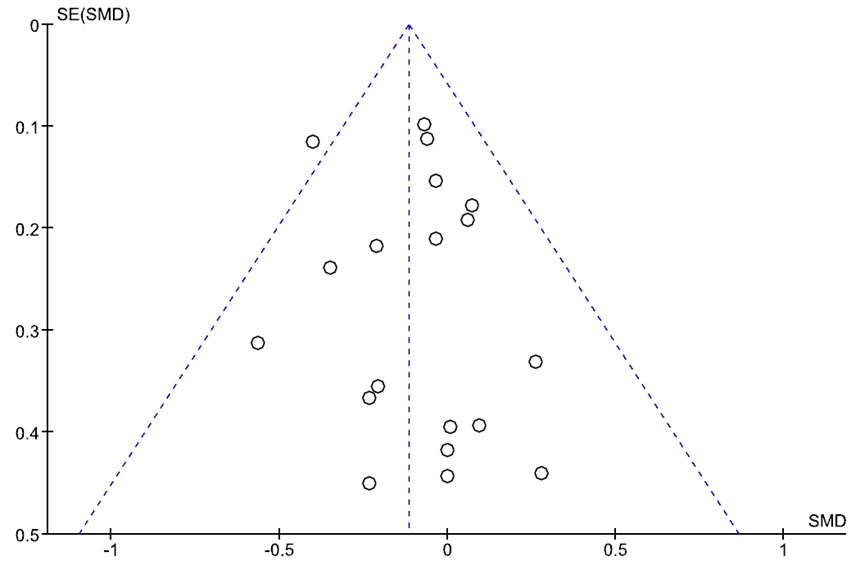


**Figure S2** – Funnel plots of comparison “Any exercise intervention versus no exercise intervention”, outcome: Total Estradiol


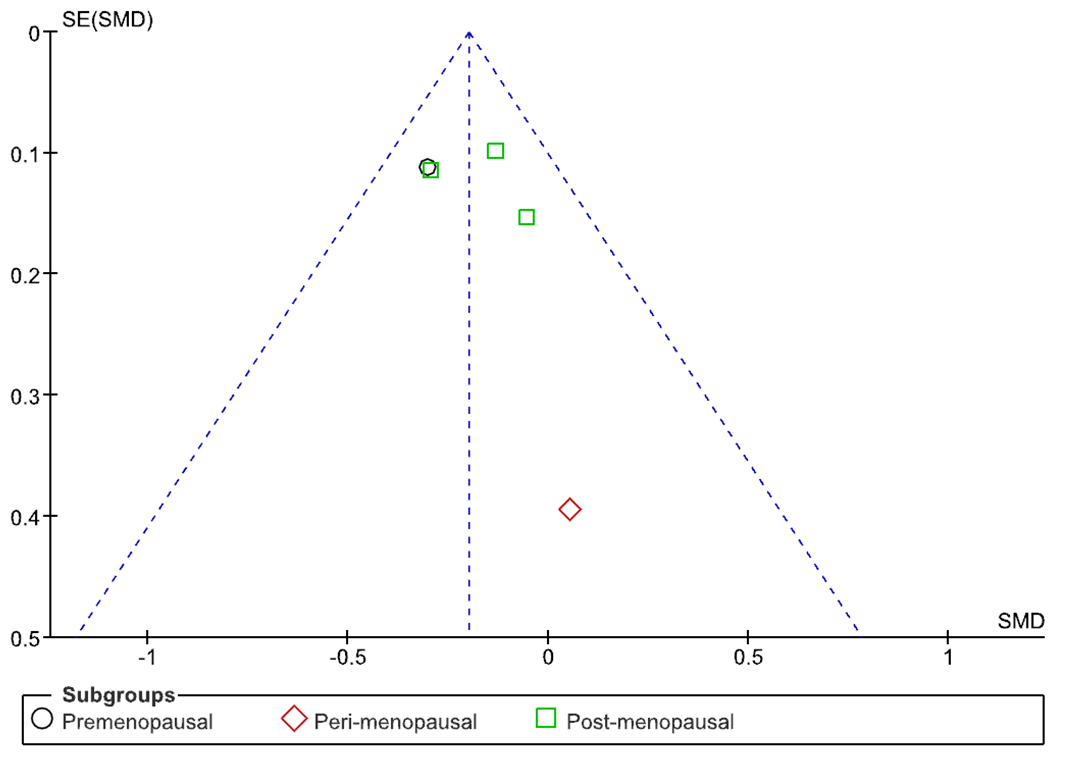


**Figure S3** – Funnel plots of comparison “Any exercise intervention versus no exercise intervention”, outcome: Free Estradiol
